# A Type I Restriction Modification System Influences Genomic Evolution Driven by Horizontal Gene Transfer in *Paenibacillus polymyxa*

**DOI:** 10.3389/fmicb.2021.709571

**Published:** 2021-08-03

**Authors:** Ziyan Chen, Minjia Shen, Chengyao Mao, Chenyu Wang, Panhong Yuan, Tingzhang Wang, Dongchang Sun

**Affiliations:** ^1^College of Biotechnology and Bioengineering, Zhejiang University of Technology, Hangzhou, China; ^2^UMR 9198 Institut de Biologie Intégrative de la Cellule (I2BC), Gif-sur-Yvette, France; ^3^Key Laboratory of Microbial Technology and Bioinformatics, Hangzhou, China

**Keywords:** *Paenibacillus polymyxa*, SMRT sequencing, type I restriction-modification system, PpoAI, horizontal gene transfer

## Abstract

Considered a “Generally Recognized As Safe” (GRAS) bacterium, the plant growth–promoting rhizobacterium *Paenibacillus polymyxa* has been widely applied in agriculture and animal husbandry. It also produces valuable compounds that are used in medicine and industry. Our previous work showed the presence of restriction modification (RM) system in *P. polymyxa* ATCC 842. Here, we further analyzed its genome and methylome by using SMRT sequencing, which revealed the presence of a larger number of genes, as well as a plasmid documented as a genomic region in a previous report. A number of mobile genetic elements (MGEs), including 78 insertion sequences, six genomic islands, and six prophages, were identified in the genome. A putative lysozyme-encoding gene from prophage P6 was shown to express lysin which caused cell lysis. Analysis of the methylome and genome uncovered a pair of reverse-complementary DNA methylation motifs which were widespread in the genome, as well as genes potentially encoding their cognate type I restriction-modification system PpoAI. Further genetic analysis confirmed the function of PpoAI as a RM system in modifying and restricting DNA. The average frequency of the DNA methylation motifs in MGEs was lower than that in the genome, implicating a role of PpoAI in restricting MGEs during genomic evolution of *P. polymyxa*. Finally, comparative analysis of R, M, and S subunits of PpoAI showed that homologs of the PpoAI system were widely distributed in species belonging to other classes of Firmicute, implicating a role of the ancestor of PpoAI in the genomic evolution of species beyond *Paenibacillus*.

## Importance

Horizontal gene transfer (HGT) is an important force. The restriction modification (RM) system is the innate immune system which limits incorporation of mobile genetic elements (MGEs). Nevertheless, the impact of RM systems on HGT-driven genomic evolution remains unclear in many bacteria. Our previous work uncovered the presence of RM systems in *Paenibacillus polymyxa*, a widely used growth-promoting, biocontrol, and antibiotic-producing bacterium. Here, through genomic and genetic approaches, we identified a pair of reverse-complementary DNA methylation motifs and showed the function of their cognate RM system PpoAI. Furthermore, the average frequency of the methylation motifs in MGEs was lower than that in the genome, implicating that PpoAI had restricted MGEs in the past. Identification of homologs of components of PpoAI in phylogenetically distant bacteria implicates that the ancestor of PpoAI could influenced HGT-driven genome evolution in species beyond *Paenibacillus*.

## Introduction

Horizontal gene transfer (HGT) is an important driving force of bacterial genomic evolution (Gogarten et al., 2002; Frost et al., 2005; Treangen and Rocha, 2011; Syvanen, 2012). Bacteriophage infection, conjugation, and natural transformation are the three main mechanisms that may lead to HGT (Ochman et al., 2000; Soucy et al., 2015). A bacteriophages is able to integrate its own DNA into the bacterial genome and coevolve with the host in the form of prophage (Nadeem and Wahl, 2017). An integrative and conjugative element (ICE) can be transferred across species *via* conjugation and inserted into bacterial genomes (Johnson and Grossman, 2015). Through transformation, exogenous DNA can be pulled into host bacteria and recombine with the genomic DNA (Dubnau and Blokesch, 2019). Genomic evolution *via* HGT can be accelerated by transposable elements which “jump” in genomes or plasmids of the same or different host bacteria (Sun, 2018). Compared with spontaneous mutation, acquiring exogenous genes strikingly increase the speed of bacterial genomic evolution (Gogarten and Townsend, 2005). The transfer of antibiotic resistance genes (ARGs), for example, contributes to the rapid development of multidrug resistance (MDR) (Forsberg et al., 2012; Mathers et al., 2015; Wang and Sun, 2015; von Wintersdorff et al., 2016; Zheng et al., 2017; Sun et al., 2019).

Nevertheless, not all genes acquired through HGT are beneficial. To reduce the risk, many bacteria have evolved adaptive (i.e., clustered regularly interspaced short palindromic repeat (CRISPR)-Cas system) (Deveau et al., 2010; Terns and Terns, 2011; Sorek et al., 2013), and/or innate immune systems (i.e., *R*estriction-*M*odification system) which are able to degrade non-self DNA (Vasu and Nagaraja, 2013; Koonin et al., 2017). A typical RM system normally contains a methyltransferase (MTase) for DNA modification and a restriction nuclease (REase) for DNA restriction. RM systems have been classified into four types (Roberts R. J. et al., 2003). The type I RM system is consisted of a specificity subunit (S) for recognizing a specific DNA motif, two DNA methyltransferase subunits (M) for catalyzing the methylation reaction, and two restriction endonuclease subunits (R) for DNA cleavage (Murray, 2000). The MTase is composed of two M and one S subunits while the REase contains two R subunits, in addition to two M and one S subunits. The S subunit normally contains two target recognition domains (TRDs) which are linked by a central conserved region (CCR) (Gubler and Bickle, 1991; Kim et al., 2005). Cleavage of DNA by the REase occurs at variable distance from the cognate DNA motif for recognition. The type II RM system is consisted of separate REase and MTase, and the corresponding motif is identical for DNA methylation and cleavage (Pingoud et al., 2014). The type III RM system is quite similar to the type I system in that both contain M and R subunits, but S subunit is absent in the type III RM system (Rao et al., 2014). DNA cleavage takes place ∼30 bp away from the DNA recognition motif. The type IV RM system has only REase that cleaves foreign DNA with methylation at the same site as the recognition motif (Loenen and Raleigh, 2014). In addition, some bacteriophages have orphan DNA methyltransferases which help them avoid degradation by the cognate REase (Murphy et al., 2013).

The interplay between RM systems and HGT has been well documented (Thomas and Nielsen, 2005; Labrie et al., 2010; Mruk and Kobayashi, 2014). In *Staphylococcus aureus*, fewer target sites for a RM system in plasmids and bacteriophages implicate the impact of target site distribution on the evolution of populations in this species (Roberts G. A. et al., 2013). Nevertheless, there remains a lack of evidence from bacterial methylome data which reveal the consequence of coeffect of RM system and HGT on genomic evolution in many bacteria (Oliveira et al., 2014). As a plant growth-promoting rhizobacterium, *Paenibacillus polymyxa* has been used as a soil inoculant in agriculture and horticulture by mechanisms including nitrogen fixation, phosphate solubilization, degradation of environmental pollutants, and producing antibiotics or lytic enzymes (Grady et al., 2016; Wang et al., 2018). It is also an antibiotic producer which biosynthesizes the last-resort antibacterial drug polymyxin (Choi et al., 2009; Shaheen et al., 2011; Galea et al., 2017) and antifungal drug fusaricidin (Li and Jensen, 2008; Yang et al., 2018). Our previous work revealed the presence of RM systems in *P. polymyxa* (Shen et al., 2018). However, molecular basis of RM systems and their DNA methylation motifs have yet to be characterized. Single-molecule real-time sequencing (SMRT) not only allows greater read lengths, permitting accurate *de novo* genome sequencing and easier genome assembly (Roberts R. J. et al., 2013) but also provides detailed information of epigenetic modification of nucleosides, making it possible to evaluate the status of methylome of a particular strain (Davis et al., 2013; O’Loughlin et al., 2015; Zautner et al., 2015; Vincent et al., 2018). In this study, we applied SMRT sequencing in identifying DNA methylation motifs as well as their cognate RM system in *P. polymyxa* ATCC 842. We also explored potential impact of the RM system on genomic evolution driven by HGT in this species. Our finding that frequencies of the methylated DNA motif in mobile genetic elements (MGEs) were lower than that in the genome implicates that genomic evolution driven by HGT was influenced by the RM system in *P. polymyxa*. The RM systems belonging to PpoAI family could impose a selective pressure favoring MGEs containing fewer targeting DNA motifs.

## Results

### SMRT Sequencing and Annotation of the Genome

**FIGURE 1:**
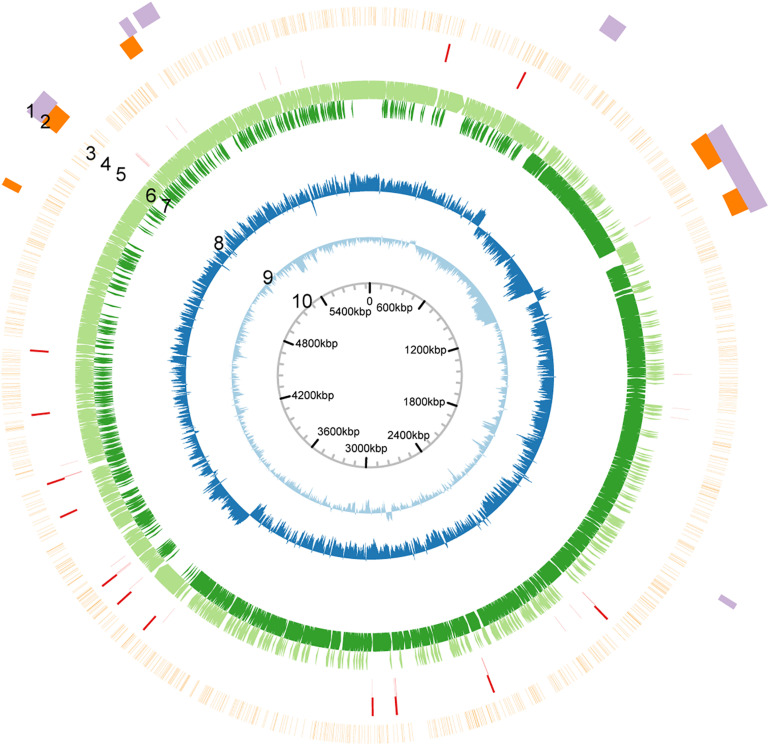
Circular map of the genome of *P. polymyxa* ATCC 842. Based on analysis of sequences of the genome of *P. polymyxa* (refer to the Supplementary Material). Circular map of the genome was drawn. From outside to inside: 1, genome island; 2, prophage; 3, density of methylation; 4, rRNA-encoding gene; 5, tRNA-encoding gene; 6, ORF in positive-sense strand; 7, ORF in negative-sense strand; 8, GC-skew; 9, GC content; 10, scale.

The genome of *P. polymyxa* ATCC 842 was sequenced by using SMRT sequencing. This resulted in 82,421 continuous long reads (CLR) with an average (total) length of 11,724 base pairs (bp) (Supplementary Figure 1). From the CLRs, 129,188 subreads (i.e., individual fragments) of an average length of 7,454 bp were extracted. Using the Pacific Bioscience *de novo* genome assembly algorithm (HGAP v. 2.0), we obtained a single 5.97-Mb contig with an average 128-fold coverage and a confidence score > 99.99% (Supplementary Figure 2). The genome sequence of *P. polymyxa* ATCC 842 obtained with SMRT sequencing is highly similar to what was originally documented in the NCBI database and less similar to that of E168 (Supplementary Figure 3). Whereas, the G + C content of the contig was 45.1%, slightly higher than that of the contig (44.9%) of *P. polymyxa* ATCC 842 originally deposited in the NCBI database (Jeong et al., 2011). The distribution of GC contents and GC skew in the genome is depicted in Figure 1. Annotation was performed with the NCBI Prokaryotic Genome Annotation Pipeline, showing a total of 5,414 coding sequences (CDSs), in which over 1,300 CDSs were not annotated in the previous study (GenBank ID: CP024795; Supplementary Table 1). A number of CDSs are classified into groups of substance transport and metabolism, according to the COG analysis^1^ (Supplementary Figure 4) and the KEGG analysis^2^ (Supplementary Figure 5). Besides, by using the antibiotic resistance genes database (ARDB) (Liu and Pop, 2009), 148 ARGs were predicted (Supplementary Table 2). Minimum inhibitory concentration (MIC) test showed that *P. polymyxa* ATCC 842 was hypersensitive to erythromycin and vancomycin (MIC < 1) and moderately sensitive to tetracyclin, kanamycin, and chloramphenicol (Supplementary Table 3).

Our SMRT sequencing analysis also revealed the presence of a 45.5-kb plasmid in *P. polymyxa* ATCC 842 (named pATCC842 hereafter). The distribution of GC contents and GC skew of the plasmid is depicted in Supplementary Figure 6. In pATCC842, 38 CDSs were predicted (Supplementary Figure 7 and Supplementary Table 4). CDS2 is supposed to encode a protein homologous to ParM, a segregation protein which provides the force for driving copies of the plasmid to the end of a bacterium. CDS4 is supposed to encode a replication-relaxation protein. CDS6 is homologous to TcpC, which is structurally homologous to the periplasmic region of VirB8, a component of the type IV secretion system from *Agrobacterium tumefaciens* (Porter et al., 2012), and therefore considered to be required for conjugative transfer. Sequences of three plasmids (i.e., pPPM1a, pSb31l, and pSC2) of *P. polymyxa* were previously deposited in the NCBI database. By using a nucleotide comparison software (i.e., progressiveMauve) (Darling et al., 2010), the sequence of pATCC842 was compared with those of pPPM1a, pSb31l, and pSC2. Interestingly, the sequence of pATCC842 showed little homology to sequences of pPPM1a and pSC2 and moderate homology to pSb31l (Supplementary Figure 8). We then performed an iterative tBLASTx of pATCC842 against the NCBI nucleotide database by using BLAST pairwise alignments. The result showed that pATCC842 was most similar to the sequence of pHD05 from *Paenibacillus* sp. IHBB 3084 (44% sequence coverage, 90% identity) (Supplementary Figure 8), implicating that pATCC 842 and pHD05 may share the same ancestor.

### Horizontally Transferred Genetic Elements in the Genome

(1) Genomic islands (GEIs) are discrete DNA segments, which is considered to be acquired from distantly related organisms and sometimes differ among closely related strains (Juhas et al., 2009). Six GEIs, ranging from 45.6 to 203.7 kb were identified in the genome of *P. polymyxa* (Table 1). Homologous fragments of these GEIs were found in several subspecies of *P. polymyxa* (Table 1), implicating that these subspecies could exchange DNA during evolution.

**TABLE 1:** Genomic island statistics.

**ID**	**Start**	**End**	**Length (kb)**	**Origin**	**Query cover**	**Identity**
GEI-1	552999	598631	45.632	*P. polymyxa* SQR-21	98%	99%
GEI-2	897043	1100769	203.726	*P. polymyxa* M1	12%	93%
GEI-3	2005201	2019060	13.859	*P. polymyxa* CF05	100%	99%
GEI-4	5058763	5117549	58.786	*P. polymyxa* HY96-2	17%	99%
GEI-5	5338561	5360592	22.031	*P. polymyxa* HY96-2	58%	99%
GEI-6	5375007	5422477	47.470	*P. polymyxa* SC2	1%	88%

(2) Insertion sequence (IS) is a short DNA sequence that functions as a simple transposable element. It is often smaller than other transposable elements and encodes only proteins involved in transposition. By using the IS search tool ISsaga (Varani et al., 2011), 78 different ISs belonging to 18 IS families were identified (Supplementary Table 5). Among them, ISs of IS3, IS4, and IS5 families are most common (Table 2), each with a frequency of more than 10 times in the genome. Origins of these ISs are extremely diverse (Table 2 and Supplementary Table 5), indicating that IS can mediate DNA exchange across bacterial species, even those in distant phylogenetic relationship.

**TABLE 2:** Statistics sample display of IS.

**Contig**	**Sequences producing significant alignments**	**IS family**	**Origin**	**Score (bits)**	***E*-value**
CP024795	ISBsu1	IS3	*Bacillus subtilis*	91.7	1.00E-14
CP024795	ISBth19	IS1595	*Bacillus thuringiensis*	85.7	7.00E-13
CP024795	ISErh1	IS3	*Erysipelothrix rhusiopathiae*	83.8	3.00E-12
CP024795	ISBpl1	IS5	*Bacteroides plebeius*	67.9	2.00E-07
CP024795	ISHaha5	IS110	*Halobacillus halophilus*	65.9	6.00E-07
CP024795	ISBma2	IS1182	*Burkholderia mallei*	61.9	1.00E-05
CP024795	ISPaen2	IS5	*Paenibacillus* sp.	60	4.00E-05
CP024795	ISBth4	IS4	*Bacillus thuringiensis*	60	4.00E-05
CP024795	ISBce7	IS4	*Bacillus cereus*	58	2.00E-04
CP024795	KIS	ISNCY	*Lactobacillus delbrueckii*	56	6.00E-04

(3) The CRISPRs, widely distributed in bacteria and archaea, can guide Cas proteins to the targeted exogenous DNA (e.g., phage DNA) for degradation (Terns and Terns, 2011; Sorek et al., 2013). Direct repeats (DR) is a hallmark of a CRISPR sequence. The transcript of a premature CRISPR RNA (crRNA) can be recognized and cut by the Cas protein at the DR site. With the CRISPR finder online tool (Grissa et al., 2007), 12 CRISPR sequences were predicted (Table 3). The number of spacers of the CRISPR sequences, which are acquired from foreign DNA, ranges from 1 to 14 (Table 3). CRISPRs can also be found in 10 other *P. polymyxa* strains, and the number of spacers ranges from 1 to 26. The most frequent DR_consensus sequence “GTCGCACTCTGTATG(G)AGTGCGTGGATTGAAAT” can also be found in four other *Paenibacillus* species (Supplementary Table 6). Whereas, no *cas* gene homolog has been predicted in *P. polymyxa*.

**TABLE 3:** Predictive statistics and description of CRISPR.

**ID**	**Start**	**End**	**No. spacers**	**Dr_consensus**
CRISPR_1	48581	48878	5	CAGCACCGACACCAGATCCGGTACCAAC
CRISPR_2	1492804	1493382	8	TTTTAATGCGTCGCACTCATATAGAGTGCGAC
CRISPR_3	1495715	1496013	4	CTTTCAATTCACGCTCCTACATAAGGAGCGAC
CRISPR_4	1504816	1505046	3	TCAATGTATGCTCCCATATAGGGAGCGAC
CRISPR_5	2146881	2147796	10	CCTGCTGGGCCAACTGGTGCTACAGGCG
CRISPR_6	3755792	3755884	1	AGTGTAAGGGCAGCTATGCTGCT
CRISPR_7	3889165	3889462	5	AGTAGCGGTGGAGCAACACCGCCAAGCG
CRISPR_8	4105418	4105690	4	CCGGCAGGCCATCAGCGCCGGTCGGTCC
CRISPR_9	4588618	4588737	1	CTAGGGCAGCTACGCTGCAGAATCAATGTT
CRISPR_10	5485682	5485775	1	ATGGGATACATTATCACGTATTTG
CRISPR_11	5660038	5661006	14	GTCGCACTCTATATGAGTGCGTGGATTGAAAT
CRISPR_12	5670117	5670840	10	GTCGCACTCTGTATGGAGTGCGTGGATTGAAAT

(4) Phages can insert its own DNA into the bacterial genome to become a prophage and replicate with the host genome (Nadeem and Wahl, 2017; Wahl et al., 2019). Six prophages were discovered by using the phage search online tool PHAST (Table 4). Three of them were predicted to have complete sets of prophage genes. Lengths and GC percentages of the six prophages vary largely, ranging from 21.9 kb to 73.7 kb, and 33.82 to 47.76%, respectively. Percentages of CDSs that match to phage genes range from 40% to 80%. To trace origins of the prophages, comparative analysis was performed and the result showed that five out of six prophages were most similar to phages in *Bacillus* or *Brevibacillus*. Of note, two incomplete prophages P2 and P3 are similar to the same phage Sundance from *Brevibacillus*, indicating that their ancestor might insert into the genome of *P. polymyxa* at two different positions of the genome during evolution. In 12 other *P. polymyxa* strains, prophages were also detected in their genomes with lengths ranging from 11.7 to 63.6 245 kb (Supplementary Table 7).

**TABLE 4:** Predicted prophages and description.

**Name**	**Completeness**	**Length**	**Position**	**GC%**	**Total protein**	**Phage protein**	**Bacterial protein**	**Most common phage**
P1	Intact	73.7 kb	892328–966098	37.10%	84	34 (40%)	7	SPG24 (*Bacillus*), NC_030903
P2	Incomplete	21.9 kb	1036124–1058030	33.82%	23	15 (65%)	1	Sundance (*Brevibacillus*), NC_028749
P3	Incomplete	42.6 kb	1048161–1090843	36.19%	20	16 (80%)	1	Sundance (*Brevibacillus*), NC_028749
P4	Incomplete	18.4 kb	4891894–4910345	47.76%	24	18 (75%)	2	BalMu (*Bacillus*), NC_030945
P5	Intact	51.1 kb	5060765–5111947	43.60%	68	37 (46%)	1	D6E (*Thermophilic*), NC_019544
P6	Intact	37.0 kb	5310732–5347812	44.34%	48	34 (71%)	3	vB_BhaS-171 (*Bacillus*), NC_030904

(5) Potential functional evaluation of a horizontal transfer gene. The expression of lysin can induce bacterial cell lysis and release bacteriophages from their original host. To evaluate the function of horizontally transferred genes, we cloned CUU60_24020 from prophage 6 (P6), which was predicted to encode a lysozyme. Expression of CUU60_24020 was controlled by an arabinose inducible promoter (Supplementary Figure 9 in the Supplementary Materials). The lysin gene from phage 21 (an active bacteriophage in *Escherichia coli*), whose product had been shown to disrupt *E. coli* cells (Wang et al., 2018), was set as the control (Supplementary Figure 10). With respect to the culture not supplemented with arabinose, expression of CUU60_24020 resulted in obvious cell growth defect both in liquid culture and on plates (Supplementary Figure 10). The result indicated that CUU60_24020 from P6 was active and therefore possibly played a role in the co-evolution of P6 with its host.

### DNA Methylation Motifs and a Putative RM System

**FIGURE 2:**
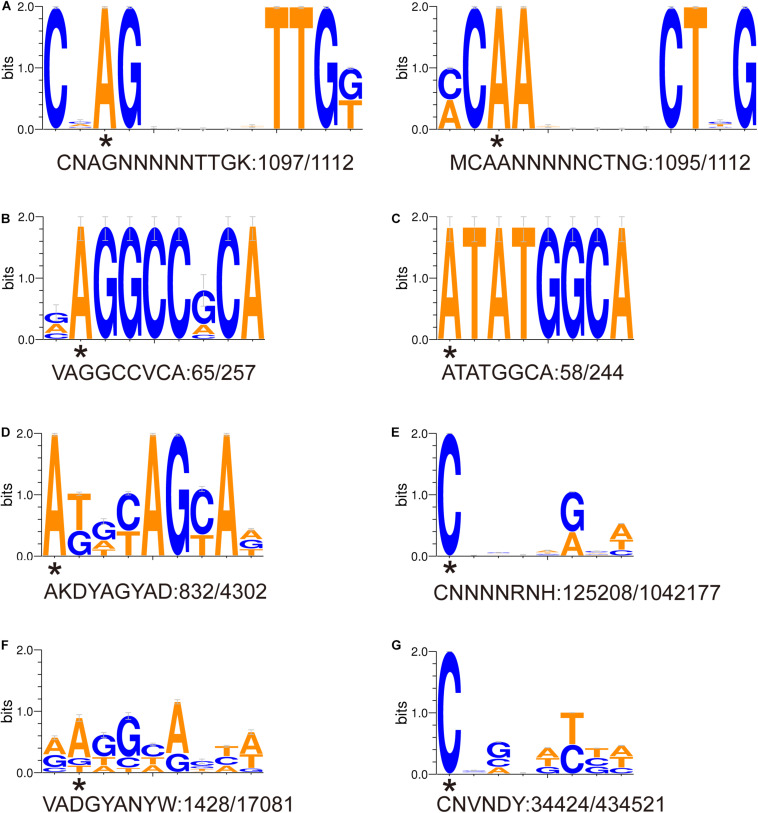
**(A-G)** Sequence logos of methylated DNA motifs. Sequence logos of methylated DNA motifs were obtained with the WebLogo 3 server and listed in Table 5. The asterisk under a particular letter indicates the methylated base. Degenerate base: B = C/G/T, D = A/G/T, H = A/C/T, K = G/T, M = A/C, N = A/C/G/T, R = A/G, S = C/G, V = A/C/G, W = A/T, and Y = C/T.

Eight DNA methylation motifs were predicted in the genome of *P. polymyxa* (Figure 2 and Table 5). The cognate RM system was analyzed with the REBASE database (personal communication with Dr. Richard J. Roberts). A pair of reverse-complement motifs a1 and a2 (CNAGNNNNNTTGK, MCAANNNNNCTNG), which can be recognized by N-6 adenine-specific methyltransferases in the two reverse complementary strands, are predicted to be recognized by a type I RM system PpoAI (Table 5). Motifs a1 and a2 are evenly distributed in the genome of *P. polymyxa* ATCC 842, without significant GC% or GC skew or ORF bias (Figure 2). CDSs encoding M, S and R subunits of PpoAI were identified in loci away from MGEs, indicating that this RM system is inherent to this strain (Table 6). By contrast, no corresponding RM systems were predicted for other putative methylation motifs that can be recognized by N-6 adenine-specific methyltransferases (b, c, d, f) or by cytosine-4-specific methyltransferases (e and g). Bacterial methylation is often complete or very close to 100%. Considering that the percentages of methylation of motifs a1 and a2 are more than 98%, they are likely to be a real methylation motif. Because only 7.92% to 25.29% of the other six putative motifs are methylated, it is questionable whether these motifs can be considered “real” methylation motifs.

**TABLE 5:** Methylation motifs in the genome of *P. polymyxa* ATCC 842.

**No.**	**Motif**	**Position**	**Type**	**% motifs detected**	**No. motifs detected**	**No. motifs in genome**	**Mean modification QV**	**Mean coverage**	**Partner motif^1^**
a1	CN 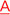 GNNNNNTTGK^2^	3	m6A	98.65	1,097	1,112	96.26	60.36	MCAANNNNNCTNG
a2	MC 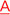 ANNNNNCTNG	3	m6A	98.47	1,095	1,112	88.98	61.48	CNAGNNNNNTTGK
b	V 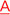 GGCCVCA	2	m6A	25.29	65	257	46.43	62.89	
c	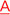 TATGGCA	1	m6A	23.77	58	244	41.16	60.88	
d	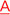 KDYAGYAD	1	m6A	19.34	832	4302	42.79.	61.75	
e	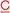 NNNNRNH	1	m4C	12.01	125,208	1,042,177	42.74	63.36	
f	V 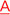 DGYANYW	2	m6A	8.36	1,428	17,081	43.58	61.83	
g	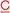 NVNDYBH	1	m4C	7.92	34,424	434,521	42.65	63.93	

*Degenerate base: B = C/G/T, D = A/G/T, H = A/C/T, K = G/T, M = A/C, N = A/C/G/T, R = A/G, S = C/G, V = A/C/G, W = A/T, Y = C/T. *QV*, quality value.*

*^1^Partner motif: the reverse complement of the motif.*

*^2^Letter in red indicates the methylated nucleoside.*

**TABLE 6:** Putative *P. polymyxa* ATCC 842 RM system.

**Enzymes**	**Locus**	**Type/subtype**	**Recognition sequence**	**% Motif detected**	**Coverage**
M.PpoAI	4583277–4584875	I/M	CN 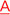 GNNNNNTTGK^1^	98.7	60.9
P.PpoAI	4578023–4581232	I/R			
S.PpoAI	4582002–4583261	I/S			

*^1^Letter in red indicates the methylated nucleoside.*

### A PpoAI-Null Deletion Mutant Is Defective in DNA Modification and Restriction

**FIGURE 3:**
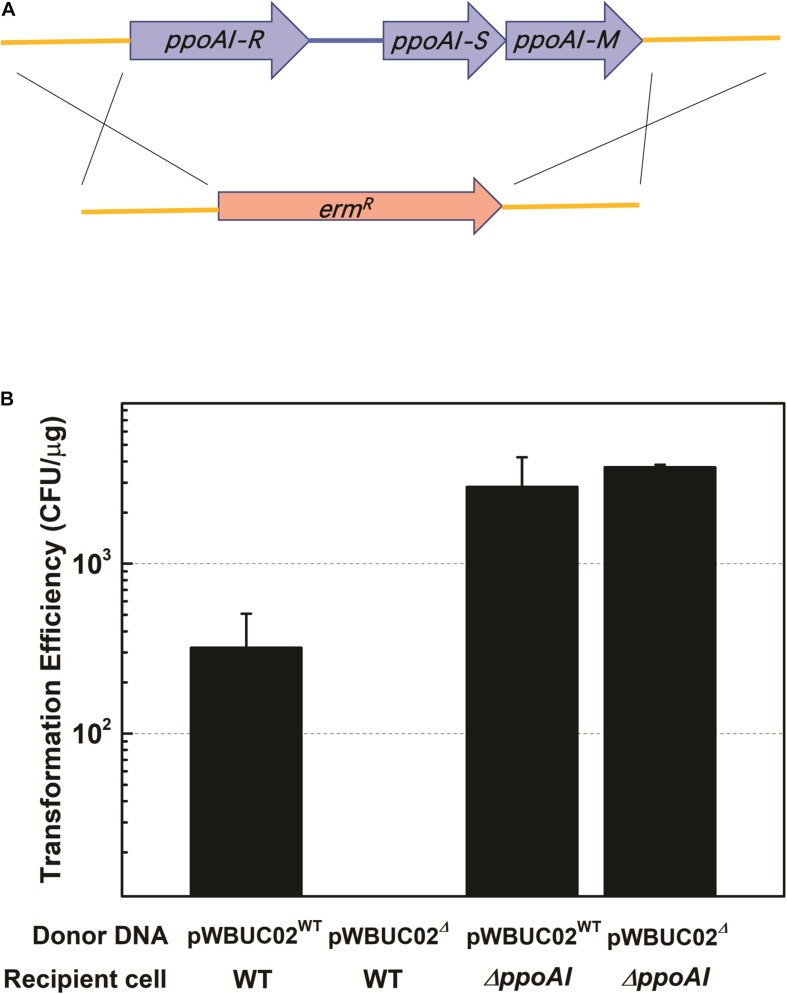
Plasmid transformation in *P. polymyxa* mutant defective in *ppoAI*. **(A)** Schematic of mutant construction through homologous recombination. **(B)** Plasmid transformation of the wild-type and the Δ*ppoAI* mutant. The experiment was performed in triplicate. Transformation efficiency was calculated as the number of transformants per microgram plasmid DNA. Data were shown as average ± standard deviation. Refer to “Materials and Methods” and Supplementary Materials for more details about the construction of the mutant and plasmid transformation of *P. polymyxa*.

To functionally characterize PpoAI, genes encoding R, M, and S subunits were replaced by a selective marker (i.e., kanamycin resistance gene) in *P. polymyxa* (Figure 3A and Supplementary Figure 11). The *ppoAI* null-deletion mutant was confirmed through PCR with a pair of primers targeting on the up- and downstream of the *ppoAI* locus (Supplementary Figure 12), as well as the flanking sequence of the *ppoAI* locus and the kanamycin resistance gene (data not shown). A plasmid named pWBUC02 containing a second selective marker (i.e., erythromycin resistance gene) was constructed to examine transformation efficiency in the *ppoAI* null-deletion mutant (Supplementary Figure 13). To characterize the function of PpoAI, pWBUC02 was isolated from the wildtype strain and the *ppoAI* null-deletion mutant, respectively, and used as the donor DNA for transformation assays. We observed that, with pWBUC02 isolated from the *ppoAI* null-deletion mutant as the donor DNA, although transformation efficiency of the *ppoAI* null-deletion mutant was ∼3.7 × 10^3^ CFU/μg, no transformants of the wildtype strain were detected (Figure 3B). The result reflected that pWBUC02 from the *ppoAI* null-deletion mutant was not methylated and therefore cleaved by the REase of PpoAI in the wildtype strain. Although pWBUC02 isolated from the *ppoAI* null-deletion mutant was unable to transform the wildtype strain, it well transformed the *ppoAI* null-deletion mutant, with a transformation efficiency of ∼3.7 × 10^3^ CFU/μg (Figure 3B). The data revealed that the *ppoAI* null-deletion mutant was unable to restrict unmethylated pWBUC02. Taken together, the plasmid transformation assay clearly shows that the *ppoAI* null-deletion mutant is defective in DNA modification and restriction.

**FIGURE 4:**
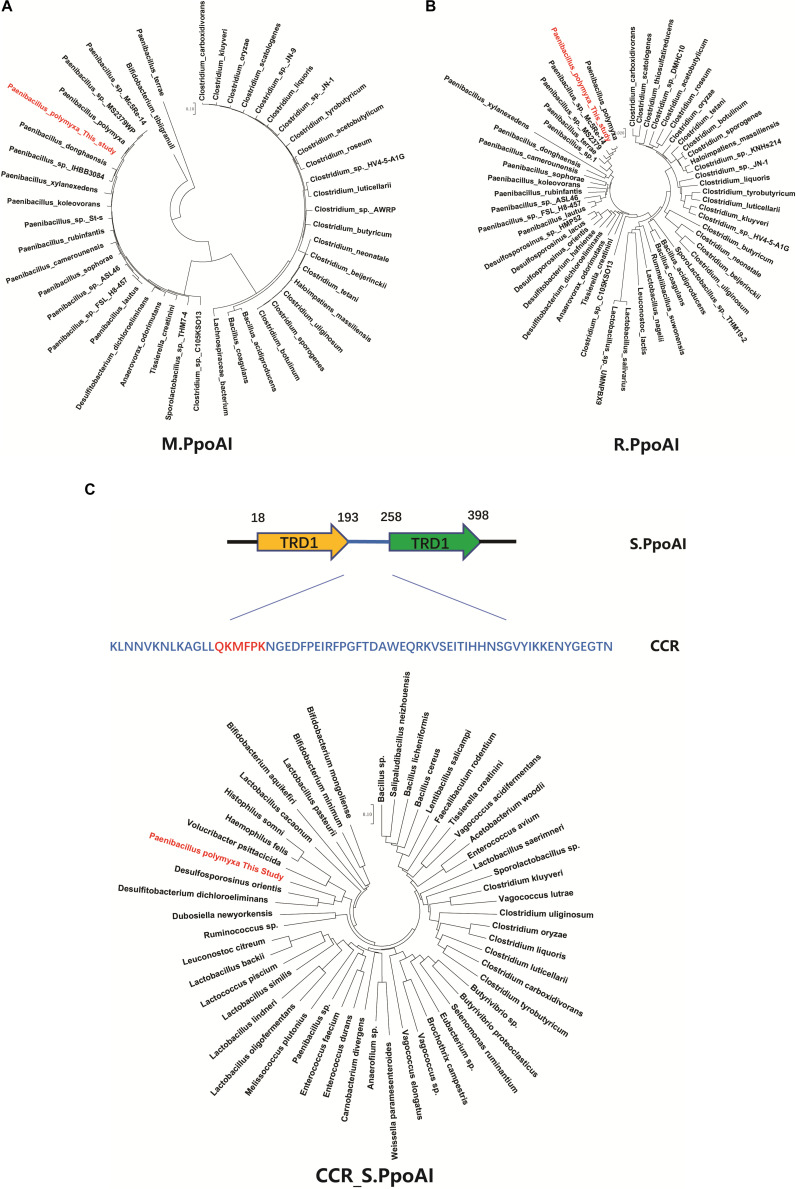
Comparative analysis of the PpoAI RM system. Homologous sequences were searched by BlastP of M **(A)**, R **(B)** and S **(C)** subunits sequence against all. The central conserved region (CCR) of the S subunit of PpoAI was predicted with the online software Proscan and homologous sequences were searched by BlastP of CCR against all. Alignment of homologs of R, M, and S subunits of PpoAI was performed by using ClustalX (refer to Supplementary Figure 14). Unrooted neighbor-joining phylogenetic trees of M.PpoAI **(A)** and R.PpoAI **(B)** were constructed with MEGA 7.0.

To further explore the phylogenetic distribution of the PpoAI RM system, we performed BlastP alignments of the endonuclease subunit (R), the specificity subunit (S), and the methyltransferase subunit (M). Homologs of both R and M subunits were exclusively found in *Firmicute* (Figure 4 and Supplementary Figures 14A,B). In contrast, no homolog of S subunit was found in the database (Supplementary Figure 14C). The region (CCR) between two target recognition domains (TRDs) was reported to be conserved (Gubler and Bickle, 1991; Kim et al., 2005). Comparative analysis of the CCR of the S subunit showed that the corresponding homologous sequences were found not only in *Firmicute* but also in species of other phyla (Figure 4C and Supplementary Figure 15). Of note, although *Clostridium* and *Paenibacillus* belong to different classes of Firmicute, homologs of R and M subunits, as well as the CCR of the S subunit, were found in a number of species in both of the two genera (Figure 4 and Supplementary Figures 14, 15).

### Correlation Between the Abundance of Recognition Motifs of PpoAI and MGEs

**FIGURE 5:**
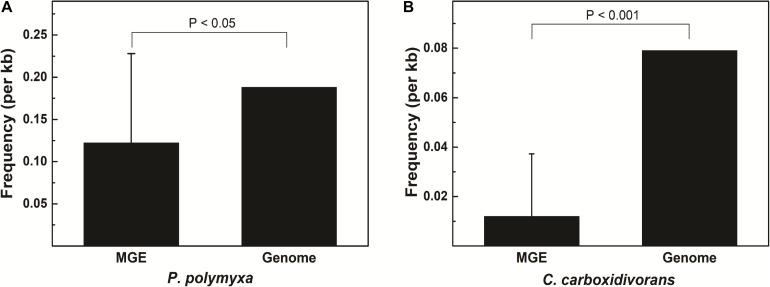
**(A,B)** Distribution of DNA methylation motifs in MGEs and genomes. Prophages were predicted with PHAST, a phage search tool. By using the online Island Viewer Genomic islands (GEIs) tool, GEIs were predicted with the integrated program SIGI-HMM and IslandPath/DIMOB, which have comparable overall highest accuracy. Refer to Table 7 for detailed information about MGEs. Adjacent predicted GEIs were manually assembled together. Statistical analysis was performed using Student’s *t*-test.

Some MGEs carry genes encoding RM systems which help MGEs parasitize in the host genome in a manner comparable with toxin–antitoxin systems (Mruk and Kobayashi, 2014). Providing that genes encoding PpoAI do not lie in MGEs (Table 6), the RM system should be evolved independent of HGT. The presence of PpoAI would restrict the transfer of MGEs carrying cognate DNA motifs, leading to MGEs containing few such motifs. To evaluate whether the PpoAI played a role in the evolution of the genome of *P. polymyxa* driven by HGT, we calculated the frequency of its cognate motif in the prephages and genome islands, as well as in the genome. The frequency of the motif of PpoAI is 0.188 per kb in the genome, which is significantly higher than that (0.122 per kb) in MGEs (i.e., pre-phages and genome islands) (Figure 4C and Table 7). Components (M, R subunits and CR1 of the S subunit) of CcaP7III in *Clostridium carboxidivorans* are highly similar to those of PpoAI (Figures 4A,B, 5). SMRT sequencing data of *Clostridium carboxidivorans* P7 have been deposited in the REBase database. In this strain, the frequency of the methylation motif of CcaP7III in the genome was estimated to be 0.0792 per kb, remarkably higher than that (0.0144 per kb) in MGEs (i.e., prophage and genome islands) (Figure 4C and Table 7). These findings indicate the RM systems belonging to PpoAI family could impose a selective pressure favoring MGEs containing fewer targeting DNA motifs.

**TABLE 7:** Frequency of the methylated motif in MGEs.

**HGT element**		**Length (kb)**	**No. motif**	**Frequency in HGT elements (per kb)**	**Ave in HGT elements^1^ (per kb)**	**Frequency in genome^2^ (per kb)**	***p*-Value^3^**
***P. polymyxa* ATCC 842**							
Prophage	P1	73.77	13	0.176	0.122	0.188	0.042
	P2	21.91	0	0			
	P3	42.68	6	0.141			
	P4	18.45	7	0.379			
	P5	51.18	7	0.137			
	P6	37.08	1	0.0270			
Genome island	GEI-1	45.63	0	0			
	GEI-2	203.73	33	0.162			
	GEI-3	13.86	1	0.072			
	GEI-4	58.79	8	0.136			
	GEI-5	22.03	1	0.045			
	GEI-6	47.47	9	0.190			
***C. carboxidivorans***							
Prophage	P1	9.0	0	0	0.012	0.079	1.2 × 10^–7^
	P2	28.1	0	0			
Genome Island	GEI-1	4.54	0	0			
	GEI-2	18.26	0	0			
	GEI-3	6.25	0	0			
	GEI-4	58.47	1	0			
	GEI-5	19.66	0	0			
	GEI-6	18.05	2	5.54 × 10^–5^			
	GEI-7	15.04	0	0			
	GEI-8	31.11	0	6.43 × 10^*m5*^			

*^1^Average frequency of the methylated motif in prophages and genome islands. ^2^Frequency of the methylated motif in the genome. ^3^The significance was evaluated by Student’s *t*-test between “Frequency in HGT elements” and “Frequency in Genome”.*

## Discussion

Our previous work revealed the presence of RM systems in *P. polymyxa* (Shen et al., 2018). In this study, a combination of genomic and genetic approaches was employed to identify a functional type I RM system in *P. polymyxa*. Through SMRT sequencing, we analyzed the genome and methylome of *P. polymyxa* (Figure 1), and showed that a pair of reverse complementary motifs 5′-CNAGNNNNNTTGK-3′ and 5′-MCAANNNNNCTNG-3′, which were methylated at the third adenine residue (Figure 2 and Table 5), were prevalent in the genome of *P. polymyxa* (Figure 1 and Table 5). Genes encoding their cognate type I RM system PpoAI were also found in the genome (Table 6). Further genetic analysis showed that the *ppoAI*-null deletion mutant lost its function in modifying and restricting plasmid (Figure 3), confirming that PpoAI indeed provides immunity against extracellular DNA.

### Could PpoAI Influence Genomic Evolution Driven by HGT Beyond *Paenibacillus*?

Horizontal gene transfer drives the evolution of genomes by introducing extracellular genetic elements (Gogarten et al., 2002; Frost et al., 2005; Treangen and Rocha, 2011; Syvanen, 2012). RM systems often limit MGEs with cognate DNA motifs (Vasu and Nagaraja, 2013; Koonin et al., 2017). In this study, we showed distribution constraints of recognition motifs in MGEs by PpoAI with regard to that in genomes (Figure 4), adding new evidence supporting direct impact of RM systems on genome evolution driven by HGT. Of note, prophage P4 contained remarkably more DNA motifs of PpoAI than the average level in the genome (Table 7). Two explanations were provided. First, the ancestor of P4 may belong to a group of bacteriophages that entered cells as the form of single-stranded DNA or RNA or expressed anti-R-M genes to avoid the constraint of RM systems (Rusinov et al., 2018). Second, the ancestor of P4, which initially contained few DNA motifs of PpoAI, entered the cell earlier than other bacteriophages, and evolved more such DNA motifs through random mutation during evolution.

A type I RM system is consisted of R (restrictase for cleaving DNA), M (methyltransferase for methylating DNA), and S (sequence-specific recognition) subunits (Murray, 2000; Loenen et al., 2014). Homologs of both R and M subunits of PpoAI were found in ∼10 species belonging to *Paenibacillus* and seldomly found in other closely related genera (Figure 4 and Supplementary Figure 14). The S subunit of a type I RM system determines the specificity of the type I RM system (Calisto et al., 2005; Kim et al., 2005; Loenen et al., 2014). The homolog of S subunit of PpoAI was not found through BlastP in NCBI database (Supplementary Figure 14C). Accordingly, the cognate DNA methylation motif was found to be unique in the REBASE database. In *Enterococcus faecium*, a type I RM system was found to be associated with subspecies separation (Huo et al., 2019). We propose that PpoAI could serve as a barrier to HGT and contribute to species separation in *Paenibacillus*. Interestingly, homologs of both R and M subunits of PpoAI were found in ∼20 species of *Clostridium* (Figures 4A,B and Supplementary Figure 14), which are phylogenetically distant to *Paenibacillus* and belongs to a different class in Firmicutes. Although S subunits are diverse, sequences of their CCRs are conserved among different species (Gubler and Bickle, 1991; Kim et al., 2005). Homologous sequences of CCR of the S subunit of PpoAI were found in several *Clostridium* species, in addition to some bacteria even beyond Firmicute (Figure 4C and Supplementary Figure 15). The frequency of its cognate DNA methylation motif also shows a distribution constraint: it was more than sixfold lower in MGEs than that in the genome (Figure 5). These observations implicate that the ancestor of PpoAI should have conferred immunity against extracellular DNA beyond *Paenibacillus*. A number of bacteria belonging to both *Paenibacillus* and *Clostridium* are valuable to agriculture, industry, and human health but intractable to genetic manipulation. Characterization of their shared RM system would provide a foundation for constructing genetic tools in strains of both genera.

### Methylation at Cytosine Is More Prevalent in the Genome of *P. polymyxa*

Single-molecule real-time sequencing revealed the presence of methylation at both adenine and cytosine sites in *P. polymyxa* (Table 5). In all, 164,207 DNA methylation motifs were detected in the genome. There are 125,208 and 34,424 copies of motif e (CNNNNRNH) and motif g (CNVNDYBH), respectively, and they both have 4-methylated cytosines (Table 5). The two motifs comprise ∼98.4% of the total copies of DNA methylation motifs. In contrast, only less than 1.6% detected methylation nucleosides locates in the rest of the six motifs with N-6 methylated adenines (Table 5). Therefore, methylation on cytosines could be more prevalent than that on adenines in *P. polymyxa* ATCC 842. Their cognate RM systems and impact to genomic evolution remain to await further investigation.

### Could the Strain ATCC 842 Serve as a Model Strain for Investigating *Paenibacillus*?

Belonging to the generally recognized as safe species, strains of *Paenibacillus* can produce a number of metabolites of agricultural, medical, and industrial values (Grady et al., 2016; Wang et al., 2018). Establishing a model strain for understanding genetics of *Paenibacillus* would provide guidance for constructing cell factories in this species. Although genetic engineering tools were available in *P. polymyxa* SC2 which seems to not have strong RM systems (Zhang et al., 2013), phenotypic variability of this strain could limit its use as a model strain (Hou et al., 2016). In contrast, *P. polymyxa* ATCC 842 was phenotypically and genetically stable from one generation to another, making it ideal as a model strain of *Paenibacillus*. Our previous work established a method for transfer and expression of exogenous genes in *P. polymyxa* ATCC 842 (Shen et al., 2018). In this study, we have successfully inactivated its chromosomal genes (Figure 3). Together, we have established a complete genetic manipulation system for expressing exogenous genes and inactivating endogenous genes in *P. polymyxa* ATCC 842. Moreover, deleting the type I RM system further increased transformability of this strain.

Nevertheless, there are still obstacles for establishing ATCC 842 as a model strain. Our previous work revealed the presence of an additional type IV RM system which efficiently degrade Dam methylated DNA in *P. polymyxa* ATCC 842 (Shen et al., 2018). Deleting the gene encoding the type IV RM system would further simplify the genetic manipulation process in *P. polymyxa* ATCC 842. In addition, we have only identified two types of plasmids (i.e., pWB980 derivative and pRN5101) which were able to replicate and two selective markers which conferred kanamycin and erythromycin resistance genes in this strain. Further expanding the genetic toolbox for *P. polymyxa* ATCC 842 would expedite the study and use of *Paenibacillus*.

### How Were MGEs Acquired in *P. polymyxa* ATCC 842

In this study, we analyzed the genome of *P. polymyxa* ATCC 842 with SMRT sequencing data. IS and GEI are hallmarks of HGT in bacteria (Syvanen, 2012; Lang et al., 2017). Comparative genome analysis revealed 78 ISs and six GEIs in the genome of ATCC 842 (Table 7 and Supplementary Table 5), indicating that HGT events in this species were frequent. Considering that most ISs and GEIs do not contain phage-like or conjugative elements, these MGEs mostly could be acquired, which is normally mediated by a conserved DNA uptake machinery (Sun, 2018; Dubnau and Blokesch, 2019). In the genome of *P. polymyxa*, a set of DNA uptake and processing gene homologs was identified (Supplementary Figure 15 and Supplementary Table 8). These genes include the *comEA* which potentially encodes the DNA-binding protein, *comEC* which potentially encodes the inner membrane channel protein, *comGA* and *comC* which could be required for the assembly of competence pseudopili. The competence pseudopilin was encoded by *comGC* in *B. subtilis*, and the ortholog of this gene was not identified in *P. polymyxa*. Assembly of the pseudopilus is mediated by the integral membrane protein ComGB in *B. subtilis* (Dubnau and Blokesch, 2019). Although neither *comGC* ortholog nor *comGB* ortholog was identified in *P. polymyxa*, two genes (*tadB* and *tadE*), which potentially encode the Flp pilus, were found in its genome. In natural transformation of *Micrococcus luteus*, Flp pilus was proposed to function as the competence pili (Angelov et al., 2015). The helicase ComFA may pull ssDNA into the cytoplasm on the expense of ATP in *B. subtilis* (Dubnau and Blokesch, 2019). However, no *comFA* ortholog was identified in *P. polymyxa*. In the cytoplasm, the incoming ssDNA is protected by the widely conserved ssDNA-binding protein DprA/Smf (Mortier-Barriere et al., 2007). Interestingly, a *dprA*/*smf* homolog was found in *P. polymyxa* ATCC 842, but absent in *P. polymyxa* SC2.

**TABLE 8:** Strains, plasmids, and primers used in this study.

**Strain, plasmid, or primer**	**Relevant genotype, primer sequence, and/or description**	**Sources/references**
**Strains**		
*P. polymyxa* ATCC 842	Type strain of *P. polymyxa*	ATCC
*P. polymyxa* ZJUT01	*P. polymyxa* ATCC 842 Δ*ppoAI*, Kan^*r*^	This study
*B. subtilis* SCK6	Erm^*R*^, 1A751 derivate, *lacA*:P*_xylA_*-*comK*	Zhang and Zhang, 2011
*B. subtilis* WB800	*nprE*, *aprE*, *epr*, *bpr*, *mpr:ble*, *nprB:bsr*, *vpr*, *wprA:hyg*	Wu et al., 2002
*E. coli* Trans5α	*supE44*, Δ*lacU169* (Φ*lacZ*Δ*M15)*, *recA1*, *endA1*, *hsdR17*, *thi-1*, *gyrA96*, *relA1*	TransGen Biotech Co., Ltd.
**Plasmids**		
pRN5101	pUC replicon in *E. coli*, Amp^*r*^ in *E. coli*, Ts replicon in *B. subtilis* and *P. polymyxa*, Erm^*r*^ in *B. subtilis* and *P. polymyxa*	Zhang et al., 2013
pWB980	pUB110 replicon, P43 promoter, Kan^*r*^	Lab reserve
pWBUC01-*egfp*	hybrid plasmid of pWB980 and pMD19-T simple; replicate in *E. coli*, *B. subtilis*, and *P. polymyxa*; express *egfp* with P43.	Shen et al., 2018
pMD19-T simple	pUC replicon, Amp^*r*^	Takara Co., Ltd.
pWBUC02	pWBUC01-*egfp* derivative, *egfp* replaced by an *erm*^*r*^ gene	This study
pRN5101-1	pRN5101 with the knock-out type l RM system homologous arm	This study
**Primers**		
P1	5′-gtattattcaagggctttttcgg-3′	This study
P2	5′-ctatgatagtttccaaatttgccg-3′	This study
P3	5′-gagtatgctcctcaattgagt-3′	This study
P4	5′-ctacctcatcatgatccttgc-3′	This study
P5	5′-cggcaaatttggaaactatcataggggccagtttgttgaagatta-3′	This study
P6	5′-actcaattgaggagcatactcggagaagttaataaatacgtaaccaa-3′	This study
P7	5′-caaggatcatgatgaggtaggtgaccaaacaggaaaaaacc-3′	This study
P8	5′-ccgaaaaagcccttgaataatactcggcttaaaccagttttcgc-3′	This study
P9	5′-gtttatggcggtgtagatgt-3′	This study
P10	5′-tggcttctgataaagcggg-3′	This study
P11	5′-cctagcacaatcagacttgc-3′	This study
P12	5′-caacttgggaatttaatttccac-3′	This study
P13	5′-ggagacatgaacgcgtgtgctctacgaccaaaac-3′	This study
P14	5′-cccgggtaccgagctcgagccctttcgtcttcaagaat-3′	This study
P15	5′-tcgagctcggtacccg-3′	This study
P16	5′-cgttcatgtctccttttttatg-3′	This study
P17	5′-gtaaaacgacggccagt-3′	This study
P18	5′-caggaaacagctatgac-3′	This study
P19	5′-ggagatatacatatggctagcatgcgcaaaatatcaaaagcg-3′	This study
P20	5′-ggagatatacatatggctagcatgcgcaaaatatcaaaagcg-3′	This study
P21	5′-aaagccatgacaaaaacgcg-3′	This study
P22	5′-gttccctactctcgcatggg-3′	This study

Although DNA uptake machinery is well conserved in bacteria, the mechanisms of competence induction for natural transformation are extremely diverse (Aas et al., 2002; Ogura et al., 2002; Claverys et al., 2006). In gram-positive bacteria, the pheromone stimulates the two component system which activates the transcription regulator for expressing late competence genes (including DNA uptake and processing genes) (Ogura et al., 2002; Claverys et al., 2006). Because pheromones and proteins involved in the competence signal transmission are often species specific, conditions for inducing natural transformation varies among different bacterial species (Ogura et al., 2002; Claverys et al., 2006; Shanker et al., 2016). It would be interesting to explore the condition for inducing natural transformation of *P. polymyxa* in the future study. Spontaneous plasmid transformation and cell-to-cell plasmid transfer without the assistance of DNA uptake machinery and conjugative machinery have been repeatedly documented in bacteria (e.g., *E. coli*) (Sun et al., 2006, 2009; Wang et al., 2007; Sun, 2016, 2018; Hasegawa et al., 2018). It is unclear whether these unconventional DNA transfer mechanisms are involved in the evolution of the genome and the plasmid in *P. polymyxa*.

In conclusion, we first analyzed the genome of *P. polymyxa* with SMRT sequencing, uncovering frequent HGT events in the genome and the presence of an originally unappreciated plasmid. Next, we identified a pair of reverse complementary DNA methylation motif and genes encoding their cognate RM system PpoAI in the genome. Genetic analysis confirmed a role of PpoAI in protecting *P. polymyxa* against extracellular DNA. Furthermore, we found that the frequency of recognition motifs in MGEs was lower than the average level, implicating that PpoAI influenced incorporation of extracellular DNA into the genome of *P. polymyxa*. Wide distribution of homologs of component of PpoAI in different classes of the phylum Firmicute implicates important role of its ancestor in the evolution of genomes of other species. Taken together, our work would not only reveal the impact of the RM system on bacterial genome evolution but also establish a model strain of *Paenibacillus* for gene function analysis and cell factory construction.

## Materials and Methods

### Genomic DNA Isolation and Sequence Analysis

Genomic DNA of *P. polymyxa* was isolated with the bacterial genomic DNA isolation kit (Takara Biotech Co., Ltd.) and sequenced and assembled according to PacBio guidelines (refer to Supplementary Materials for the detail). DNA methylation was detected by using the RS_Modification_and_Motif_Analysis protocol within SMRT Portal v2.30, with a standardized *in silico* false-positive error < 1%. The motifs with a mean modification quality value (QV) higher than 50 and a mean coverage of > 100 × were validated as being modified^3^. The consensus sequences of all motifs were depicted as sequence logos that were obtained by the WebLogo 3 server^4^ (Crooks et al., 2004). Antibiotic resistance genes were predicted with ARDB^5^ (Liu and Pop, 2009). RM systems and the DNA motifs with methylation were analyzed with Rebase^6^ (Roberts et al., 2015). The insertion sequence was predicted with the ISsaga online server^7^ (Varani et al., 2011). Prophages were predicted with an online phage search tool (PHAST)^8^ (Zhou et al., 2011; Arndt et al., 2019). CRISPR finder^9^ was used for identifying CRISPRs (Zhu et al., 2016). Genomic islands (GIs) were predicted with Island Viewer^10^ (Bertelli et al., 2017). Structural regions of the S subunit of PpoAI were predicted with the online tool Uniprot^11^.

### Strains, Plasmids, Primers, Media, and Growth Conditions

All strains, plasmids, and primers used in this study were listed in Table 8. *E. coli* and *P. polymyxa* were cultured in Luria-Bertani medium containing 0.5% (w v^–1^) yeast extract, 1% (w v^–1^) typtone, and 1% (w v^–1^) NaCl or on LB agar plates supplemented with or without appropriate antibiotics. The 10 × Spizizen minimal medium was prepared as follows: 4% (w v^–1^) (NH4)_2_ SO_4_, 18.3% (w v^–1^) K_2_HPO_4_⋅3H_2_O, 6% (w v^–1^), KH_2_PO_4_, 1% (w v^–1^), Na_3_C_6_H_5_O_7_⋅2H_2_O, and 0.2% (w v^–1^) MgSO_4_⋅7H_2_O. *B. subtilis* was cultured in GM1 containing 0.8% (w v^–1^) glucose, 0.04% casein hydrolysate and 0.1% yeast extract supplemented with 10% (v v^–1^) of the 10 × Spizizen minimal medium, or GM2 medium containing 0.8% (w v^–1^) glucose and 0.02% casein hydrolysate supplemented with 10% (v v^–1^) of the 10 × Spizizen minimal medium, or on LB agar plates supplemented with appropriate antibiotics. All strains were incubated with shaking at a speed of 180 rpm under 37°C or 30°C. Plasmids were isolated from bacteria with the SanPrep column plasmid mini-preps kit (Axygen Biotech Co., Ltd.). To isolate plasmid from *B. subtilis* and *P. polymyxa*, lysozyme (4 mg/ml) was added to the cell resuspension solution and incubated at 37°C for 1 h.

### Plasmid Transformation of *E. coli*, *B. subtilis*, and *P. polymyxa*

Plasmid transformation of *E. coli* was performed according to the documented chemical transformation method (Cohen et al., 1972; Sun et al., 2013). When the cell culture was grown in LB medium, to an OD_600_ of 0.4, cell pellets were collected by centrifugation, washed with 100 mM CaCl_2_ solution twice, and resuspended in 100 mM CaCl_2_ solution on ice. Plasmid was transferred into the competent *E. coli* cell with heat shock for 90 s, and transformants were screened on LB plates supplemented with ampicillin (100 μg/ml).

Plasmid transformation of *B. subtilis* was performed by using the Spizizen’s method (Anagnostopoulos and Spizizen, 1961). A single colony of *B. subtilis* was inoculated into 5 ml GM1 medium. The overnight grown culture was transferred to 5 ml of fresh GM1 at an inoculum of 0.2% (v v^–1^). The bacterial culture was further grown to the end of exponential stage (1∼2 h) before being transferred to 5 ml of the GM2 medium. After 1∼2 h of incubation in GM2 supplemented with or without 1% xylose, plasmid DNA was added to the cell culture before screening on plates supplemented with kanamycin (50 μg/ml) or erythromycin (25 μg/ml).

Plasmid transformation of *P. polymyxa* was performed by using our previously established method (Shen et al., 2018). A fresh single colony of *P. polymyxa* was inoculated into LB medium and incubated at 37°C overnight. The overnight grown culture was inoculated into 50 ml of fresh LB supplemented with 20 mM sorbitol. Exponentially growing cells were collected by centrifugation and washed with the precooled solution A three times, followed by resuspension in the solution B. Plasmid isolated from either *B. subtilis* or *P. polymyxa* was transformed into 50 μl aliquots of electrocompetent cells. Transformation efficiency was calculated as the number of transformants per μg of plasmid DNA. Refer to the Supplementary Materials for more details about plasmid transformation of *P. polymyxa*.

### Construction of Recombinant Plasmids and *P. polymyxa* PpoAI Null-Deletion Mutant

The temperature-sensitive plasmid pRN5101 was previously used in deleting chromosomal gene in *P. polymyxa* SC2 (Zhang et al., 2013). A derivative of pRN5101 (pRN5101-1) for deleting genes encoding PpoAI was constructed by inserting the DNA fragment containing a kanamycin resistance gene flanked by upstream and downstream of genes encoding PpoAI into the vector by using the one-step cloning kit (Vazyme Biotech Co., Ltd.). To examine transformability of *P. polymyxa*, pWBUC02 conferring erythromycin resistance was constructed by ligating the DNA fragment containing an erythromycin resistance gene from pRN5101 with linearized and *egfp*-deleted plasmid pWBUC-*egfp*. Refer to Supplementary Materials for details about construction of pRN5101-1 and pWBUC02. To construct the *ppoAI* deletion mutant, pRN5101-1 was isolated from *E. coli* DH5α and then transformed into *B. subtilis* with the Spizzen’s method as described. pRN5101-1 from *B. subtilis* was then electroporated into *P. polymyxa* and screened on LB-agar plates supplemented with erythromycin (25 μg/ml). Transformants were cultured with shaking at a speed of 180 rpm in LB supplemented with kanamycin (50 μg/ml) at 37°C. Every 12 h, 1% (v v^–1^) of the culture was inoculated into fresh LB medium containing kanamycin (50 μg/ml) and incubated with shaking at 37°C. After three rounds of inoculations, the serial diluted cell culture was spread on LB agar plates supplemented with kanamycin (50 μg/ml), followed by screening for colonies unable to grow on LB plates containing erythromycin (25 μg/ml) at 30°C. Kanamycin-resistant erythromycin-sensitive colonies were selected and examined through colony PCR with the primer pair P11–P12.

## Data Availability Statement

DNA sequence data generated in this study have been deposited at NCBI as *P. polymyxa* ATCC 842 with CUU60 locus tags (BioProject Accession: PRJNA416965, BioSample: SAMN07972645, GenBank ID: CP024795).

## Author Contributions

DS substantially contributed to the conception and design of the work. ZC, MS, CM, CW, and TW contributed to the acquisition, analysis, and interpretation of data for the work. PY and ZC are responsible for the revision and proofreading of this work. All authors contributed to the article and approved the submitted version.

## Conflict of Interest

The authors declare that the research was conducted in the absence of any commercial or financial relationships that could be construed as a potential conflict of interest.

## Publisher’s Note

All claims expressed in this article are solely those of the authors and do not necessarily represent those of their affiliated organizations, or those of the publisher, the editors and the reviewers. Any product that may be evaluated in this article, or claim that may be made by its manufacturer, is not guaranteed or endorsed by the publisher.

## Footnotes

1
http://www.ncbi.nlm.nih.gov/COG

2
http://www.genome.jp/kegg

3
https://github.com/PacificBiosciences/Bioinformatics-Training/wiki/Methylome-Analysis-Technical-Note

4
weblogo.threeplusone.com/create.cgi

5
https://card.mcmaster.ca/download

6
http://rebase.neb.com

7
http://issaga.biotoul.fr/ISsaga

8
http://phast.wishartlab.com/

9
http://crispr.u-psud.fr/Server/CRISPRfinder.php

10
http://www.pathogenomics.sfu.ca/islandviewer

11
https://www.uniprot.org
