# Supplementary material for: A Type I Restriction Modification System Influences Genomic Evolution Driven by Horizontal Gene Transfer in Paenibacillus polymyxa
Source: Front Microbiol. 2021 Aug 3;12:709571. doi: 10.3389/fmicb.2021.709571 (PMC8370563; doi:10.3389/fmicb.2021.709571)
Supplement: Supplementary file 1 [file Data_Sheet_1.PDF]

SUPPLEMENTAL MATERIALS

A type I restriction modification system influences genomic evolution driven by horizontal gene transfer in *Paenibacillus polymyxa*

Running title: A RM system affects genomic evolution driven by HGT

Dongchang Sun[1]*, Ziyan Chen[1], Minjia Shen[1], Chengyao Mao[1], Chenyu Wang[1], Tingzhang Wang[2], Huan Chen[2], Juanping Qiu[1]

1. College of Biotechnology and Bioengineering, Zhejiang University of Technology, Hangzhou, Zhejiang 310014, China

2. Key laboratory of Microbial Technology and Bioinformatics of Zhejiang Province, Hangzhou, 310012, China

*Corresponding author: sundch@zjut.edu.cn

Tel/Fax: +86 0571 88320057

SUPPLEMENTAL MATERIALS & METHODS

Genomic DNA isolation, sequencing and assembly

Genomic DNA of *P. polymyxa* was isolated by following the manufacturer's instructions. The overnight grown *P. polymyxa* culture was resuspended in 20 mg/mL lysozyme and placed at 37 °C for 1 hour. Buffer BL containing 2 mg/mL proteinase K and 0.5 mg/ml RNase was added to the lysozyme-treated cell resuspension solution and placed at 56 °C for 10 minutes before adding equal amount of Buffer GB and ethanol. The mixture was transferred to spin columns for centrifugation. The genomic DNA attached to the column resin was washed with Buffer WA and Buffer WB sequentially and resolved in the double distilled water.

Genomic DNA libraries have been prepared following the PacBio guidelines and sequenced through a SMRT cell using Pacific Biosciences RSII sequencing technology (Menlo Park, CA, USA). High molecular weight DNA was mechanically sheared to an average size distribution of 20 kb fragment in a Covaris g-TUBE (Covaris, Woburn, MA, USA). After shearing, the DNA size distribution was checked using a Fragment Analyzer (Advanced Analytical Technologies, Ames, IA, USA). 5 μg of the sheared gDNA was repaired using polishing enzymes. The SMRT bell template was created through a blunt end ligation reaction followed by exonuclease treatment. The SMRT bell template and long DNA fragments (> 7 kb) were selected with A Blue Pippin system (Sage Science, Inc. Beverly, MA, USA). The quality and quantity of the DNA library was inspected through an Agilent Bioanalyzer (Agilent, CA, USA) and a Qubit Fluorimeter (Thermo Fisher Scientific, MA, USA). By using a

single SMRT cell with P6-C4 chemistry and MagBeads, the recovered library was sequenced with the PacBio RSII platform (Menlo Park, CA, USA).

Sequence analysis

Processing of the raw SMRT sequencing data was performed by using the Pacific Biosciences SMRT Analysis System (version 2.3, Menlo Park, CA, USA). SMRT Portal was used for preliminary assembly of the reads which reflect the general information of the sequenced genome. The genome sequence obtained by SMRT (ATCC 842a) was compared with the originally documented genome of ATCC 842 (ATCC 842b) and *P. polymyxa* E68 (1). The assembled genome was annotated by using the prokaryotic annotation pipeline system of NCBI. Open reading frames (ORFs) and functions of the corresponding proteins were analyzed with the NCBI non-redundant protein sequences (NR), Clusters of orthologous groups of proteins (COG, http://www.ncbi.nlm.nih.gov/COG), Kyoto encyclopedia of genes and genomes (KEGG, http://www.genome.jp/kegg), and Swiss-Prot (http://www.ebi.ac.uk/uniprot). The plasmid was predicted with the online tool plasmid finder (https://omictools.com/plasmidfinder-tool) (2) and compared with other plasmids by using progressiveMauve (http://gel.ahabs.wisc.edu/mauve) (3).

Plasmid transformation of *P. polymyxa*

Electrocompetent *P. polymyxa* cells were prepared according to the following protocol (4). A colony from a freshly streaked plate was cultured in 5 mL of LB at 37 ℃ and 200 rpm overnight (~ 12 h). At an inoculum of 4%, the overnight grown culture was inoculated into triangle bottles with 50 mL LB liquid medium containing

20 mM sorbitol and maintained at 37 °C with shaking at a speed of 200 rpm until it reached an OD_{600} of 0.5. After incubation, cells were cooled on ice for 30 min before being collected by centrifugation at 4 °C (5000 rpm for 5 min). The cell pellet was washed three times with 30 mL ice-cold Solution A containing 0.5 M sorbitol, 0.5 M mannitol and 10% (v v^{-1}) glycerol and 30 mL ice-cold Solution B containing 0.5 M trehalose dihydrate, 0.5 M sorbitol, 0.5 M mannitol and 10% (v v^{-1}) glycerol, followed by resuspension in 1 mL ice-cold Solution B and reservation at -80 °C.

A shuttle plasmid in *Escherichia coli* DH5α was extracted according to instructions of the SanPrep Column Plasmid Mini-Preps kit (Axygen Biotech Co., Ltd). The plasmid isolated from *E. coli* was then transformed into *B. subtilis* by using the Spizizen's method (5), and isolated with the SanPrep Column Plasmid Mini-Preps kit (Axygen Biotech Co., Ltd). The plasmid from *B. subtilis* was introduced into *P. polymyxa* ATCC 842 through electroporation. Aliquots of 50 μL electrocompetent *P. polymyxa* cells were mixed with 0.25 μg plasmid DNA and the mixture was incubated on ice for 30 min, which was then transferred to cuvettes (1 mm) prior to electroporation at an electric field intensity of 1.8 kV/cm. Immediately after electroporation, 950 μL of preheated recovery medium (LB medium supplemented with 0.5 M sorbitol) was added into the electroporated cells which was then incubated at 37 °C or 30 °C, 200 rpm for ~ 4 h. Transformed cells were spread on LB agar plates supplemented with 50 μg/mL kanamycin or erythromycin (25 μg/mL). Colonies were counted after 24 ~ 36 h of incubation at 37 °C or 30 °C. Transformation efficiency was calculated as the number of transformants per μg of

plasmid DNA.

Bacterial antibiotic resistance test

Resistance of *P. polymyxa* to vancomycin, tetracycline, kanamycin, erythromycin and chloramphenicol was evaluated by using the microtiter dilution assay, with McFarland standards as a reference to adjust the turbidity of bacterial suspensions (6). *P. polymyxa* ATCC842 was grown on LB agar for 12 h at 37 °C prior to antibiotic resistance testing. Each well of the sterile plastic microtiter 96-well plates was filled with the medium supplemented with vancomycin, tetracycline, and chloramphenicol at different concentrations. The bacterial culture was grown to the turbidity of 0.5 McFarland standard before being serial diluted and inoculated to the 96-well plate containing LB broth supplemented with antibiotics of a serial concentrations. After incubation at 37 °C for 12 h, the optical density of the culture at 600 nm (OD600) was read with a microplate reader. The minimum inhibitory concentration (MIC) was defined as the lowest concentration, in μg/mL, of the test antibiotic at which cell growth was suppressed.

Plasmid Construction

To evaluate the function of a gene on the HGT element, pCUU60_24020 containing a lysin encoding gene CUU60_24020 was constructed. The DNA fragment containing ORF of CUU60_24020 was amplified from the genome of *P. Polymyxa* ATCC 842 with the primer pair P19-P20 and the PCR product was digested with *Nhe*I and *Eco*RI, followed by ligation with the vector pGLO which had been digested with the same

restriction enzymes. The ligation product was transformed into *E. coli* DH5α and the recombinant plasmid pGLO-CUU60_24020 was examined by colony PCR with the primer pair P21-P22. Sequence of CUU60_24020 was shown in SEQ. 1 below.

To construct the plasmid pRN5101-1 for deleting genes encoding the PpoAI restriction modification system, DNA fragments containing the kanamycin resistance gene and up- and down-stream of *ppoAI* were PCR amplified from pWB980 with the primer pair (P5-P6) and the genome of *P. polymyxa* with primer pairs (P1-P2 and P3-P4) respectively. The three DNA fragments were then ligated together with the one-step cloning kit (Vazyme Biotech Co., Ltd) and cloned into the linearized pRN5101 (7) which had been obtained by PCR amplification with the primer pair (P7-P8). The constructed plasmid pRN5101-1 was transformed into *E. coli* DH5α and the recombinant plasmid was examined by colony PCR with the primer pair (P9-P10). Sequences of the PpoAI encoding genes and the erythromycin resistance gene flanked by up- and down- stream homologous arms were shown in SEQ. 2 and SEQ. 3 respectively below.

To obtain a plasmid harboring a selective marker for evaluating transformability in the *ppoAI* deletion mutant, pWBUC02 conferring erythromycin resistance was constructed. The erythromycin resistance gene amplified from pRN5101 with the primer pair P13-P14 was ligated with the linearized and *egfp*-deleted pWBUC01-*egfp* which had been obtained through PCR amplification with the primer pair P15-P16.

The ligation product was transformed into *E. coli* DH5α and the recombinant plasmid was examined by colony PCR with the primer pair (P17-P18). Sequence of pWBUC02 was shown in SEQ. 4 below.

Cell lysis assay

To evaluate the function of a putative bacteriophage lysin encoding gene CUU60_24020, 10 mM arabinose was added into the liquid medium or on plates before inoculating or striking *E. coli* DH5α containing pGLO-CUU60_24020. The plasmid pGLO-lysin, which had been shown to express a bacteriophage lysin for cell disruption (8), was set as the positive control.

SUPPLEMENTAL FIGURES

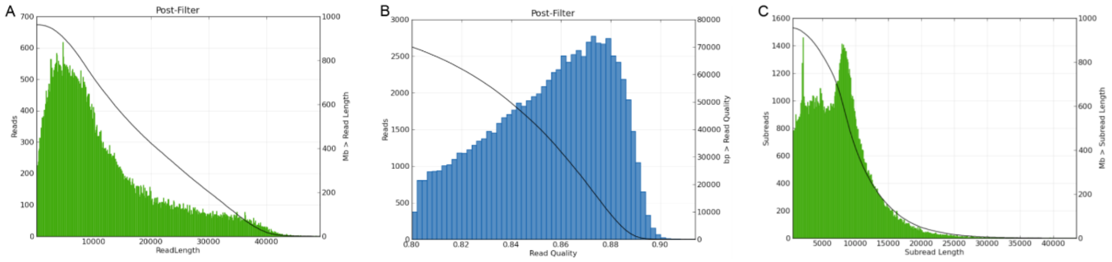

Figure S1 SMRT sequencing of *P. polymyxa* ATCC 842. (A) Read length distribution of 82421 continuous long reads (CLR) obtained from a single SMRT cell after filtering for low quality. The black line depicts the cumulated amount of bases covered by reads of a minimum size as shown on the x-axis. (B) Distribution of read quality values (1 = 100 %) for 82421 CLRs after filtering. The black line denotes the average length of reads with a quality at least as good as indicated on the x-axis. (C) Subread length distribution of 129, 188 subreads (i.e. individual fragments of CLRs).

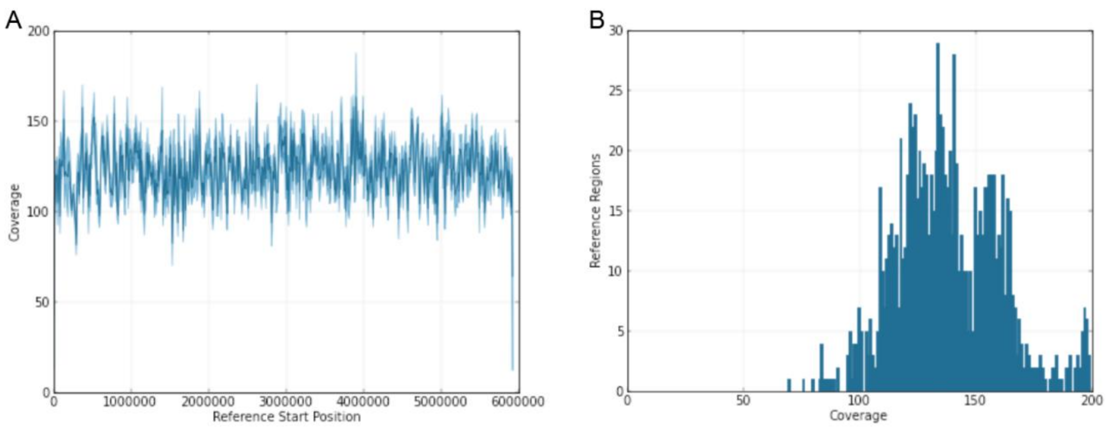

Figure S2 Reference Coverage. (A) Reference coverage (number of read base pairs per position) of the polished assembly across the contig comprising 5, 967, 522 bp. Average reference coverage is 128.19-fold. (B) Histogram of reference coverage across the assembled contig.

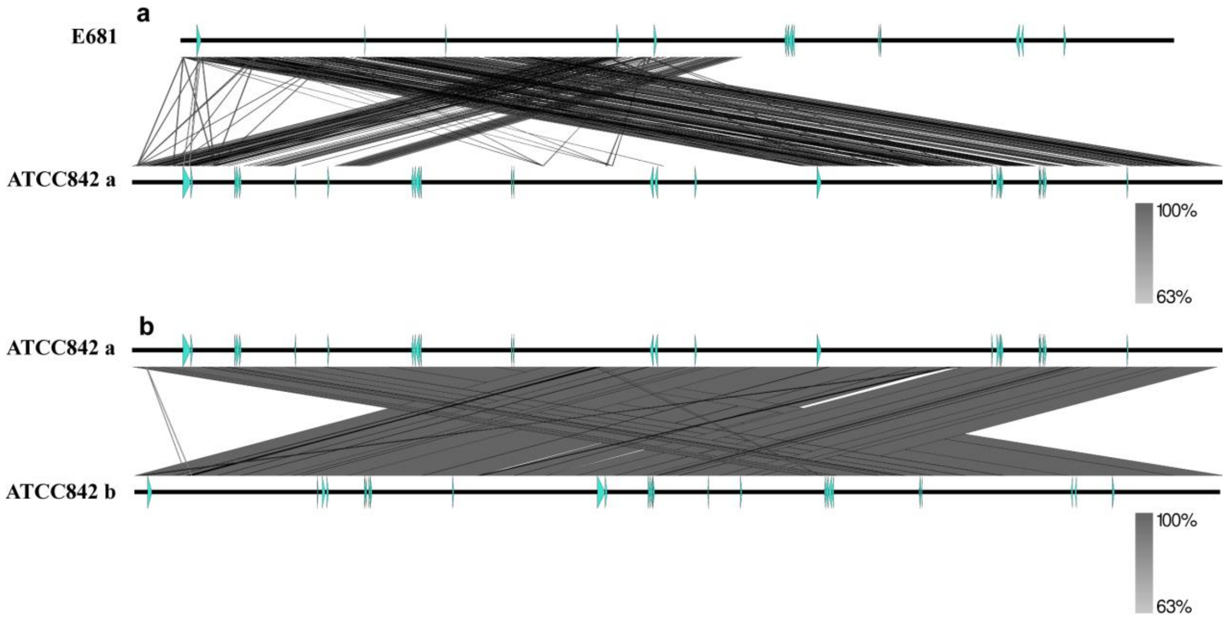

Figure S3 Comparison of genomes sequenced by SMRT and the traditional method. By using the genome comparison tool Easy-Fig, the genomic sequence of *P. polymyxa* ATCC842 obtained by SMRT (ATCC842a), was aligned with sequences of *P. polymyxa* ATCC842 (ATCC842b) and *P. polymyxa* E68 obtained by the traditional method.

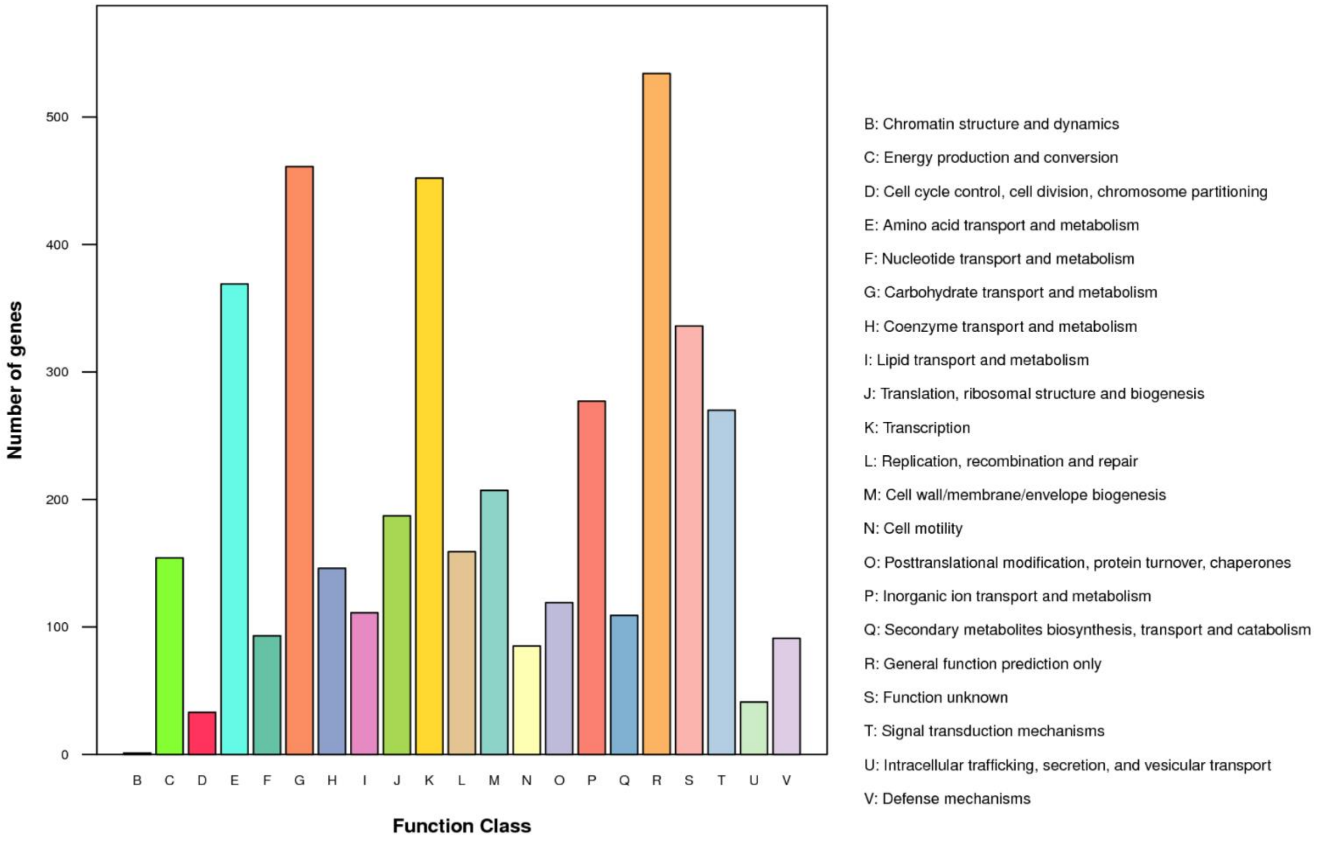

Figure S4 COG function classification of *P. polymyxa* ATCC842. The abscissa indicates the function class and the ordinate indicates the number of genes on the annotation, in which amino acid transport and metabolism (E), carbohydrate transport and metabolism (G), transcription (K), general function presiction only (R) account for a large proportion in the 21 classes.

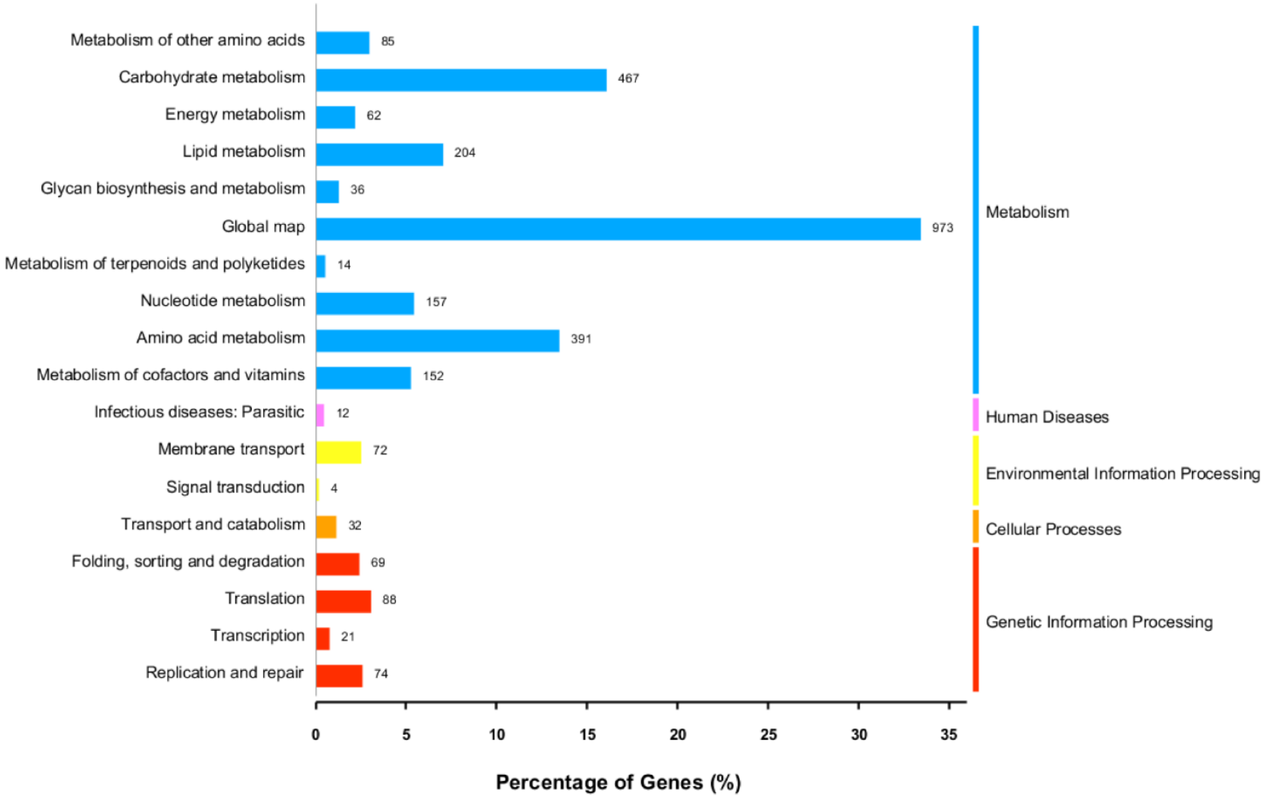

Figure S5 KEGG pathway of *P. polymyxa* ATCC842. Genes can be classified into 18 subgroups which belong to five groups: metabolism, human diseases, environmental information processing, cellular processes, and genetic information processing.

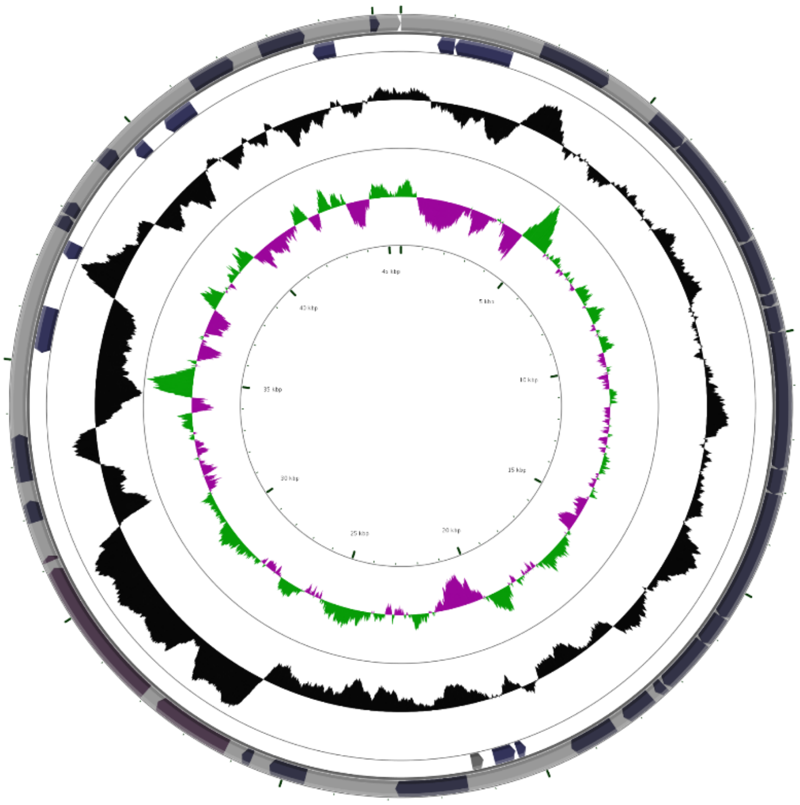

Figure S6 Contig circle diagram of pATCC842. The length of the plasmid is 45, 524 bp. The contents of the featured rings (starting with the outermost ring) are as follows. Ring 1, CDSs and rRNA; Ring 2: GC content; Ring 3: GC skew.

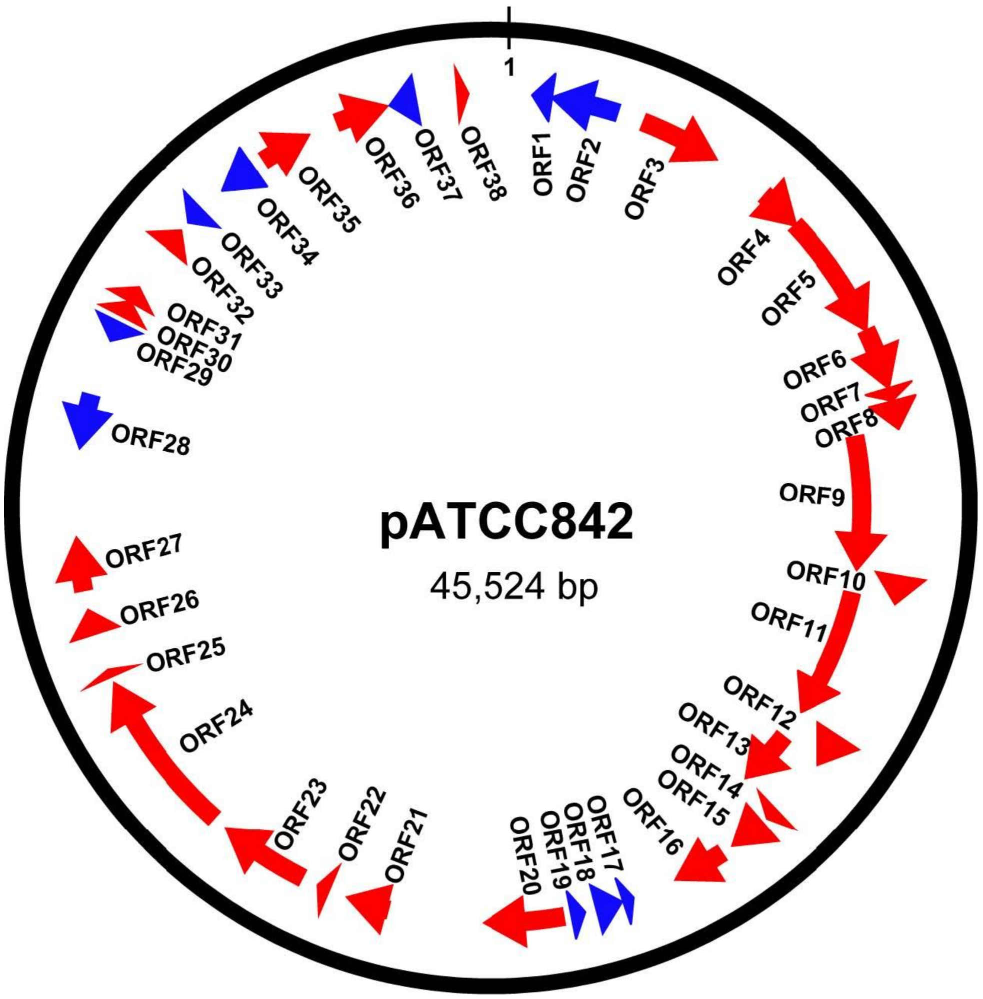

Figure S7 Genetic map of pATCC842. Coding Sequences (CDSs) are represented by block arrows. CDSs on the leading strand were marked in red and those on the lagging strand were marked in blue. The annotation of the CDSs was described in Table 1.

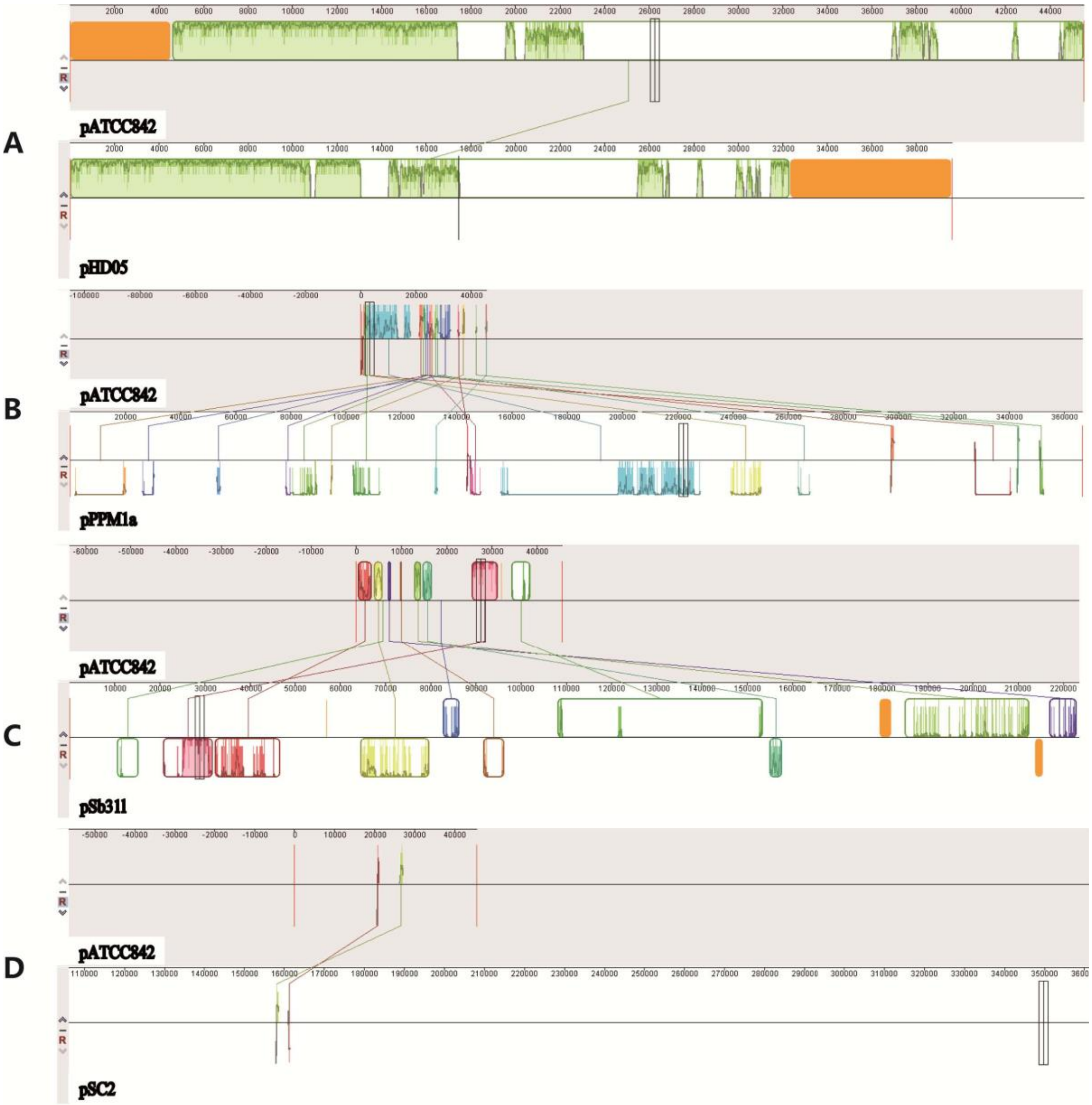

Figure S8 Comparison of sequences of pATCC842 and other plasmids from *Paenibacillus*. The sequence of 481 pATCC842 was compared with those of pHD05 (A), pPPM1a (B), pSb31l (C) and pSC2 (D), by using the 482 software ProgressiveMauve.

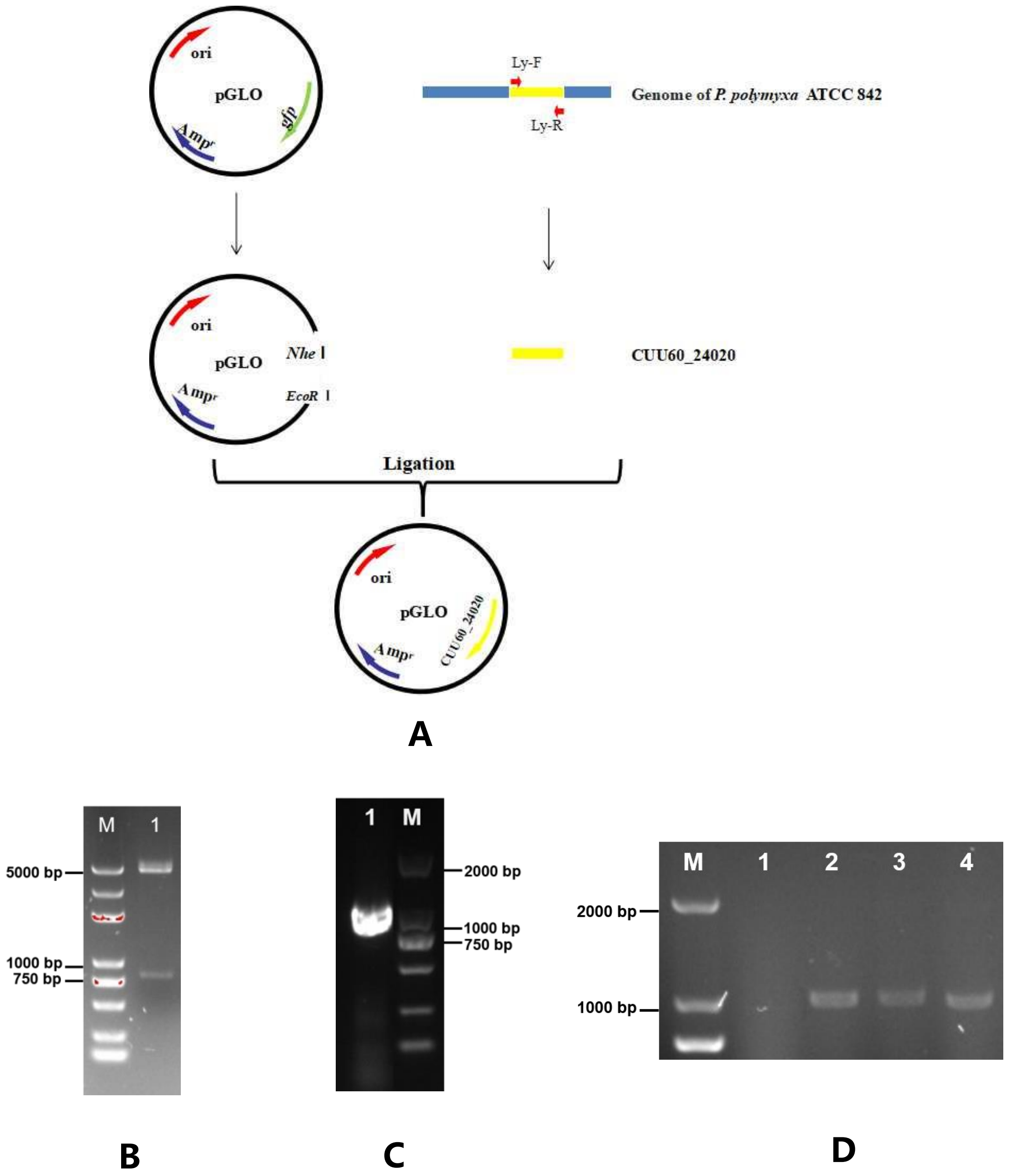

Figure S9 Construction of plasmids pCUU60_24020. The schematic diagram of construction of pCUU60_24020 was shown in (A). The vector plasmid pGLO (BioRad Accession ID: NISQOC15) was digested with *Nhe* I and *EcoR* I (B), before being ligated with the PCR product amplified from *P. polymyxa* ATCC 842 (C). The ligation product was transformed to *E. coli* DH5α and the recombinant plasmid in four transformants (lanes 1 - 4) were examined by colony PCR (D). M: DL5000 or DL2000 marker.

SEQ. 1: Sequence of pCUU60_24020

atgcgcaaaatatcaaaagcgggactaagtttaattaaaaattccgagggctgtcgcttgtatgcatacaagcctgtacccac
tgaaaaatggtggaccatcggctggggacattacggcccggacgttaagcagggcatgaccattacgcaggaacaggcg
gacgcaatgttggccgaggacatgggtaaatacgaagcttacgttaacaacccggcctacgtcccagttacggatcagctc
acacaaaaccagttcgacgcacttacaagtttttgctacaactgcggtgcaggcaatctcaaggcgctctgtaaaggtcgca
cagtcgcacagattgctgccagtatcaccaaatacgacaaggccgggggtaaggtgctagctggtctgacacggcgcag
acaggccgagcttgatctttttaacacaccgtcggattcaacggaggataaaaaggaggacaaaccagtgagccaagaaa
gagatattaacgtacccagcaagtgggcagccacagcgtgggccgaggtaacggccaacgggtattttgacgggacgca
tcctgaagcgcagatcacacgcgaggaatccgcaatcgtgattaatcggctaaggaaaaatttcctagccttgattgcaggg
gttaacggtaacgtgaccgacctggacgaacgcctgaaacagattgaatcagaaggataa

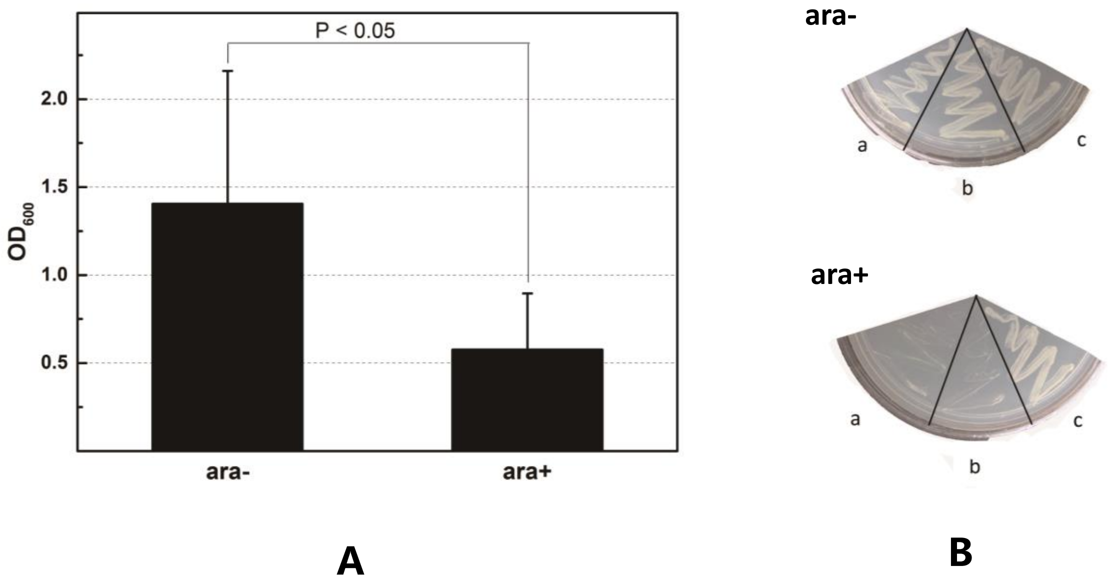

Figure S10 Effect of expression of CUU60_24020 on cell growth. *E. coli* DH5α carrying plasmid pGLO-CUU60_24020 was grown in liquid culture supplemented with or without arabinose at a concentration of 10 mM (A), or on plates that were spread with 50 μl of 2 M arabinose before striking (a: *E. coli* DH5α pGLO-lysin; b: *E. coli* DH5α pCUU60_24020; c: *E. coli* DH5α pGLO) (B).

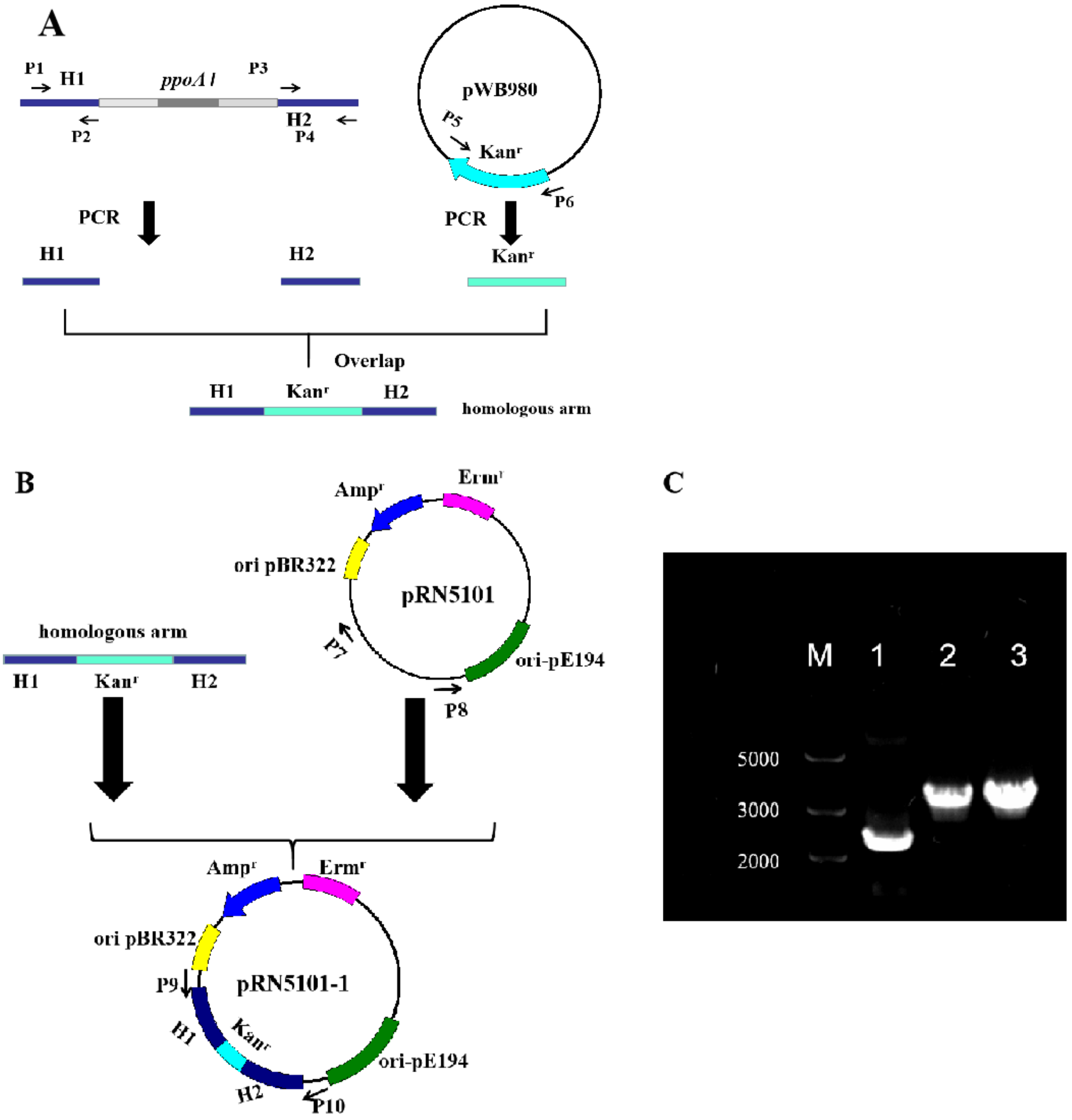

Figure S11 Construction of pRN5101-1. DNA fragments containing the kanamycin resistance gene and up- and down-stream of *ppoAI* were PCR amplified from pWB980 and the genome of *P. polymyxa* respectively, and then ligated together with the one-step cloning kit (Vazyme Biotech Co., Ltd) (A). The ligation product was then linked with the linearized vector pRN5101 obtained by PCR amplification (B). The recombinant plasmid was examined through colony PCR (C). Lane M : *Trans* 2K Plus DNA Maker; Lane 1 : pRN5101 as the template; Lanes 2 & 3 : transformants containing pRN5101-1 as templates.

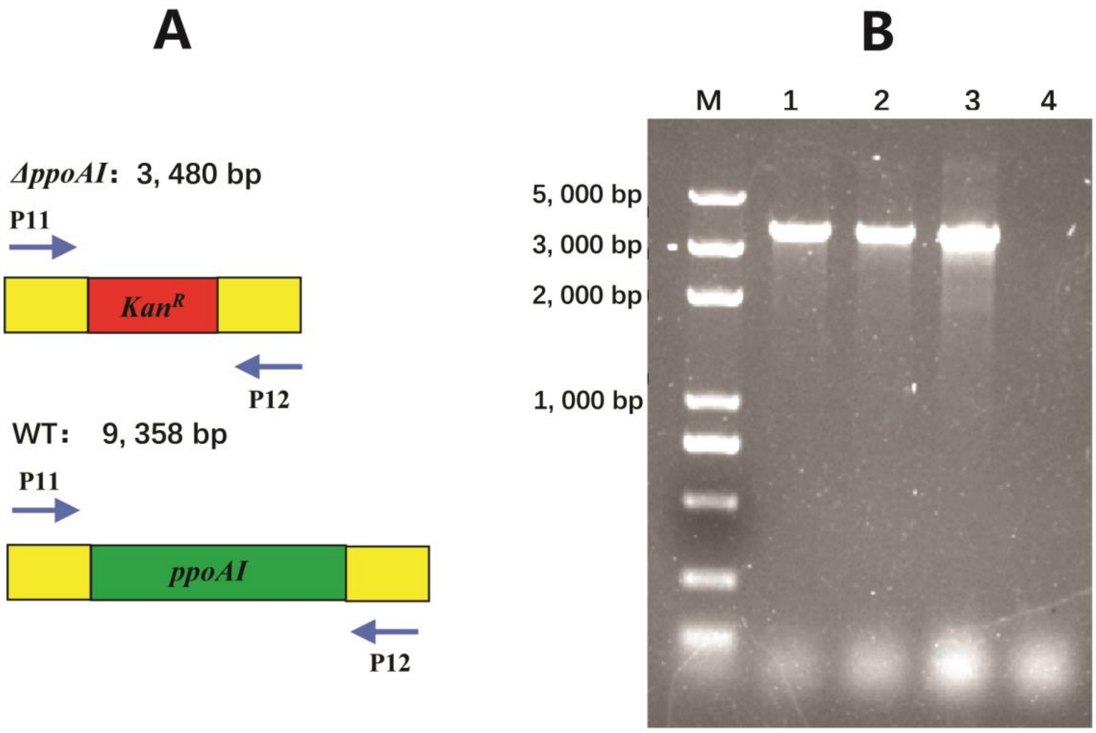

Figure S12 Examination of *ΔppoAI*. (A) Schematic of genetic structures of genes encoding PpoAI and selective marker *Kan^R* flanked with homologous arms in WT and *ΔppoAI* respectively. Corresponding sequences were shown in SEQ. 2 and SEQ. 3 below. (B). PCR examination of *ΔppoAI* and WT with the genomic DNA as the template. Lane M : *Trans* 2K Plus DNA Maker; Lane 1 ~ 3: *ΔppoAI* mutant; Lane 4: WT. Bands of expected sizes were detected in the *ΔppoAI* mutant sample. Whereas, the PCR product of the WT was too long to be amplified.

SEQ. 2: Sequence in WT (letters in red and in blue indicate homologous arms and genes encoding PpoAI respectively)

P11

5'-cctagcacaatcagacttgctagagattgttaattatataagcgaacaactttctgcaccacaagctgcaattaatcttgttgataagttagata
gggccatttcaaagttagaacaatttcctttctctggacatctctataaaaacaactttaagcttaaggatgaatttcgaatgcttgttgttgagaatta
tattgtattctacgttgtttttgatgatattgtagaaatccgacgagttatatatagtaaacgcaatcatgaaaaactactttgataataattttaatcaat
gttgattgaatagcctctcttcattgtctatctttgctacaaactcgaatagtcaaagtaataatattagctttacaataaattaggtccttggtattattc
aagggcttttcggttccatatatacacataaatatgatattgcgtaataagtaaagatcaattatggaatatttggtataaaaaatgaattgtagtatt
ttttgcctcaggtgtatagacctttgatttagttgagatatttcaatgaggtgaaaacagttggcaagaatgtattatgcagaaggcagacatattg
agagggctatttattataatagataaaaaatacaataaggttaaatatatcctttccatcgcctcgacacatgtagaaaacgtaatttttttaaaattaa
aaaatatcgattgcgttttcgaaaaaaattcttataagatacaagtatcaacaataaaagttaatttaagaaaggcacgaatttatatgaagagattag
atagtatagtagagttcgtgagtggttcaccgcaatttagaattatagaagtgtttgatgataaagcaccactttacacctattatggacaacaaga
tattgaagatgatttggttggtattgattctaatggtagtgacagcaaacaagtacgaaccttggataaggtcagtacgttatgtcaaggggatatt
gtttttagtttgatatcgggaaaatctactatggttggtgtgaagcatcagggatatctatacacgcaaaactacgttaaattagtaactgatgaaaa
aattgagtcaaaatatcttgtttacttacttaatgaagataaattcattaataaacagtttcaaatgggactacaggggtctcaagttctaaaatacac
actaaaacaagtgaaagaacttgagctaccagatctgcctataatagaaaaacagcgcataatcggtgaactctatttcaaccaattgcgacta
gaagcattgaaaattagagcggcaaatttggaaactatcatagatgaccgagaatcaatttgaaacagaattaattcaatacctgacgagtcgc
acaattgtagaacaagaacatcctgaaggaacgggtaactttgatatcaaagaatcaagtgctggctacatagtgaaaacaaaattatggaaat
atgaaccgcatattaaatctacagaacaactctggaataatttcaaggcgatcctggaacaacacaatcagaatacgcttgaccatcctttaagt
gttgtggaatttaatcaagttaagaaaattatctctgatattcagacccccctatgaagcagggcagttcttatatggtttaaatggggtttcgcaaat
cgaaatagatttagatgatggacgtcatgtgtttcttactgtgtttgatcaaaaacaaataggggctggagataccgtttatcaagtggtcaatcaa
atcaaacgaccatcagtgatcacaggaaaaggagactgtcgttttgacacaactctcttgattaatggtttaccaataattcaaattgaagaaaag
cgtgatacgcgtaatgtcgatgaagcgctcaatcagatgcagcaatatgctgcagatcatcagtatcgtgatattttctcaaccttgcaaattttgg
tggcaatgacaccaaataatgtgaagtatatggcgaatacgacggcagataaatttaataaggattttgcttttaactggcaacgcaagagtgac
aataccattgtgcgtaactggaaagaatttgctgattctatgctttcgattccaatggcgcatcaaatggctactaactacatgattttggacggaa
caaagaacaaacaaacgttgaaagtgatgcgtccgtatcaagtgtatgcgacacaaaatgtgatcgagggcttgaaacgttcagattttgaact
tggtacccataaaatcggttatatctggcacacaacgggatctggtaaaacgatcacaagcttcaaaacggcatggctggcaagtcgtatgcc
gaaagtggacaaggttgtctttgttgtggatagaattgctttaactaagcaaaccaatgaaaattacaatgcctatgatcctgatgcgacagaaga
cacatttggtagcgttcaaaacacagacaatacgactgatttgagtcgtaagcttaaaagtaaagacaacagcattattgttacttcggttcaaaa
actagataccttggtgaaacgcaaaacctttcaagctccagataagaatatcgtatttatcgtggacgaagcccatcgttcaactggcggtgact
cattcaagaatatccaaaaagcttttaagaaatctgcttgggtgggctatacgggaacaccgatgtttgatgaaacgaccactggtcttcgaaca
gaagatatttttggtccactattacatgcatataccattcgtgaagcgattgctgaccgtaatgtgttaggattcaaggtggattttgagacaacaat
tgacgaaaaacaaatgaaagaaaaatatcttcctgcgttctaccgtgaacgctatcccaactggaatgaagaacgaattcaagagaaaatcaa
caatctctcccaggaagatatggatgatgctgttgaacccagtttctacgatgaaaattcggatcatatcaaattagtagtgcaagacattttcaaa
aattggcgtaatcgttctaatgaaggcaagtataatgctttgttcacaactcatgtgggtggtggtaaagcaagtacgccaatggcaatgatgtat
tttaaggagtttcaacgtgtcaacgaagaaaataagaagaatggcggacaaacattaaaagttgcggtgacatttagtcaaaacacgtcaaaca
atgatagtatgcttgccacaaatcaaggtttgcaccatgctatgacagcttataatactgaatttggcacatcttttgggatggatgatgtttctggct
atacccaagacgtgacttcacgcttaaataaatcggtcactgatcaaaatttcctcgatttggttattgtggttgaccaattattgactggttttgatg
cacctgaacttaacacgctttatgtggatcgaaccttaaaaggcgcggggttaattcaagcttattcaagaacaaatcgtattgcagatatgcaa
gagaagccatgggggcgtgtggtgaattaccgctggcctgctcaaaatgagaagttaatgaataaagcacttgctgtttatgcgaataaagact
cagccattctgtctgaagaagatcaacgtgaatctaatcagaaagatggtatcattgccaaaccatttgaagatgtatttaatgaagtgaaagaa
gtggtggggaagctaggtagcttaacgactgaatttcaacagttgcctccatctgaaaagaaaaaggacgagatgctcgatctgctccgtgaat
ataacagagggatggcaaaattaaagcaatatgattccgatgaagttgatggtgaaaaagttggatttaactatgatgatcgagatgagttaata
gagaagctgggaatgacttcagatcaagagaaaatgttaacaaccgttttaacgaatgaactaaagtcacatattgctaaagagaagaaaatcc

ctatctatcaaatcgaactgcggatgacacatgtgaaggatattagagttgactatgattatcttactgaactcgtggaacgattattgaacgaagtccacgaaggcaaagatgaagaagccaaagagacacaagaaaaaattaatcaatttgccaatgggttggatgatcgtaactatgcaactaaaattatgaatgcagctactgcaattatcaaaggtcacttcccacctgcaggctctaacttcacgtatcccgtgaagctgagtgacagtgaacatattatccagcaagcgaacaacgtcagcctggatcgaaggtttcttgatttccgagtgaagtggggaattactgatattatcacaagtgctcaaatgcgagagttgtttagtcgccatcgctacggcttacaagacttggatgatacgggtcaaatccgtgatataatcgctcaagccagttctgactataagacacttgctcatgatgaagaagtgcagtcattatctaaaattaaatatcgcaacggtttgcgcgacgcaatttacgaactagcggatgaattggcagaaagttaatctagttgaaattgtccaataaggggttcagatgggagattgtgatgaaccaattacaatttttaatgtatgatgccgatgaaaaagtggttagtgcagaaattgcacataccactcaacatggagcaatagaaggaaaggcacagacaaacgctgttaaatattacaatctggatgccattatatcagttggctatcgtgtaaattcacaaaaggcaacaaagttccatatatgggcaacggatgtcttaaaagattacatccaaaaaggttttaaaattgatgttgaacgtatgaaacaaggtgagcatgtttttagcaaggattacttcagtgagctgattgaaaccgttcgttccattcgagcaagtgaacgacggatatggcaacaaatatcagatgtatttgcggaaatttcatacgactatgacaggaacgcagagatcaccaagaagttttatgcaacagttcaaaataagtttcattatgccattacaggcaaaacggctgctgaaattgcttatgacagtgccgataaaggcaaagatcatatggggcttacttcttggaagaattcgcccgagggtctacgaaaagcattgatgaattcttaacatttaaatcgctatgaaatcttggatggaaatgggcgcatttcacaaagtgcagctaaggaaaaggctattcacgagtataaagaattcaacaagcatcaaaaaatagtttctggttttgatagagtcattaagaccatacagggagagcagaaatgaacgaaaacaaagaaaatacacctagaataagatttcatggattcactgatgcttgggaacagcgtgagttgggggaggttatagacaatcagtataatggtcaaaccccttccagagcgaattctagtttttggaatggagatattaattggctttctagtggtgagttaaacagaggtgttgtttctaaaacaatcgaaaaaattacagagaacggaaggaaaaatgctaatctaaaaattgttccaaagggtacatttgtgatggcaataaccggtttagaagcagctggaactcggggtaactgtgcaaaattaggaatagacactactttaaaccaatcatgtatggcactgtatcctaacgaaaaattacttaattcatcgttcttgtttcaatggtatcgaaaagtaggcgaagaatacggattacaatacacacaaggaacaaagcaacaaagctacaacgctgaattaataaaaaaacttccgattattttaccttctgtaagtgaacaaaaactaattgccgactttttgacaccctagaccacctcatcacccttcatcagcgtaaattaaataacgtgaaaaatttgaaggctgggttgcttcaaaaaatgtttccgaaaaatggtgaagattttcccgaaattcgtttccctggattcactgacgcttgggaacagcgtaaggtatcggaaatcactatacaccacaactctggagtctatatcaagaaagaaaattatggcgaaggaacgaacataattggtgtcggtaatatgtacgacagcgacattattgacggaaaaatttatcgtttggctcccgtaaaggacgacaagtttattcttaatgaaaatgacttgatttatggagagtcttctcttgttccagaggggattgctcgtgtgatgtgcgtcgatgaaaatggctctgggactgcgtttgcgtggcatactcgtagattcaaaattgataacgaagtggcgaattctcactttataactttgctgctcaactacaacaaatccgttcgccagtacctcatgaatgtatcaactcaaaccgctttgacgggaataacaacggagggctttttggggcaatgattatgtttccaaacattattgaacaaaaaaagaatttctaatttttcaaacaacttgacaacctcatcacccttcatcagcataagttggaacacttgcaagaacagaagaaagcactactacaacaaatgtttatctaggaggaaaataacaatatgagcaataatctgcaaactattacaacaaaactatgggcgatggcaaatgagcttcgtggaagcatggatgctgcagaatataagaactacattttagcctttatgttttaccgttatctgtcagaacatcaagaacagtatttagttaaaaataatgttattgatgttgccgttggtgaatcaattaataaagcttatttagaacaagcaattggtgacgatttgaatgactacctagaagatatttcttcaagtttaggttatgccattgcaccgcttgatacttgggaatctttagttcataagattgataatagcatggtgattccaagtgattatcagactatttttgataacttcaatcgaaacgctgaattaaatagagaagctgcgaaagattttcgtggcgtatttaacgatattaatcttggggattcacgtctaggttcttctacaaatgaacgtgcgaaatcacttaaccgaattgtaaagcttgttcatggcattgaatacaagggtgaagataacaaggatattcttggtgcaatctatgaatacctcatcggtcaatttgcagcaagtgcaggtaaaaaaggtggagaattctacactcctcacgaagtttctaaagttttggctaaaatcgtaaccgatggtgtagaagagtcggaacatactttctcgctgtatgacccgacttgtggatctgggtcattgctgttaacggttcaaggtgagctgcctggtggaaataaacccggggcgattaaatttttggtcaagaaaagaatacgacaacatacaacttggctcgtatgaacctgatgatgcatggtgtttcatttaataatatgactctgtctaatgctgacacacttgaaagcgattggcctgatggaccagatgcccaaggaactgaccatccacgctcatttgatgccgtggttgcgaaccctccttattctgctcattgggacaatagcgaaacgaaactcaaagatccacgttttagggaatatggtaaactcgctcctagaactaaggcggattatgcattcattttacacagcttgtatcatctgaatgatactggaacaatggcgattgtattaccgcatggtgtcttgttccgtggtgcagcagaaggtgcgattcgccaaacgatcattgagaaaaactatcttgatacagttataggattacctgccaacttgttttacggcgtatccatcccaactacgattttagtattcaagaaaaatcgtaaaacaaaagacatcttgtttatcgatgcaagcaatgaatttgaaaaaggtaaaaatcaaaacaaacttactgataaaaacatcaataagattatcgaaacgtaccgaaatcgtgtagatgttgataaatatgctcacgtggcatcacttgaagaaatcaaagaaaatgagtttaacttaaatattcctcgctacgtggatacgtttgaagaagagcaagtcattgaccttgccgaagtgagtaagcttttagagcaagataaacaagaaatcgctgaacttgaagctaaaatcaatgaacagctgaaaattttgggtattcatgtgtaggagt

atgctcctcaattgagtgtgatatactcatttagatgtctatgcttaacatatactgccgtttgaactacgttgcttaaatatgttatatgtgaagaaga
agcaaggacatgaacaggttagatttaccattttgggaccaaagatttgtcctggtatttatagaggagtttttgatatgacgaataatttttggcgt
gatttgccacgacctttttttatactggcaccgatggaagatgtgacggatgttgtgtttcgccatgtcgtaagtgaagcagccagaccggatgtg
ttttttacggagtttgcgaatacagagagttattgtcacccggaggggaacaaaagtgtacgcgggcgtttgacttttacagaggatgaacagc
ccatggtggcccatatctggggagataagccggaatatttccgtgaaatgagcatcggtatggcgaaagaaggcttcaaaggcatcgatatta
atatgggttgtcccgtagcgaatgtagcccagaatgggaagggaagcggtctgatctgccgtcccgaaatcgcagcggatatcattcaggcc
accaaagccgggggactgcccgtgagtgtaaaaacaaggctcggtttcactaccgtggacgaatggcgcgactggttgacccatattttgaa
acaagacattgtgaatttgtccattcatctgcggacaagagaggaaatgagcaaagtagacgcccactgggaactgattccggagattaagaa
acttcgtgatgaggtggcaccagatacactgctgaccattaacggagatattccggaccgtcagaccggcttgaagctcgctgagcaatatgg
agtggatggtattatgattgggcgtggtattttccagaatccatttgcgtttgagaaggagcggaaggaacatagcagtgaagaattgcttgaac
tgctgcggcttcatttagatctccatgatcaatattcagcgcaggagccacgttcgttcagccctctcgcccgcttcttcaaaatatatgtccgtgg
attccgggggcaagtgaactcagaaacagcttaatgaacgccaaatcaacaagtgaagtgcgtacattgcttgatgagtttggaagcaagg
atcatgatgaggtagaggaacgtggaaattaaattcccaagttg-3’

P12

SEQ. 3: Sequence in *ΔppoAI* (letters in red and in black indicate homologous arms and kanamycin resistance gene respectively)

P11

5’-cctagcacaatcagacttgctagagattgttaattatataagcgaacaactttctgcaccacaagctgcaattaatcttgttgataagttagata
gggccatttcaaagttagaacaatttcctttctctggacatctctataaaaacaactttaagcttaaggatgaatttcgaatgcttgttgttgagaatta
tattgtattctacgttgtttttgatgatattgtagaaatccgacgagttatatatagtaaacgcaatcatgaaaaactactttgataataattttaatcaat
gttgattgaatagcctctcttcattgtctatctttgctacaaactcgaatagtcaaagtaataatattagctttacaataaattaggtccttggtattattc
aagggctttttcggttccatatatacacataaatatgatattgcgtaataagtaaagatcaattatggaatatttggtataaaaaatgaattgtagtatt
tttttgcctcaggtgtatagacctttgatttagttgagatatttcaatgaggtgaaaacagttggcaagaatgtattatgcagaaggcagacatattg
agagggctatttattataatagataaaaaatacaataaggttaaatatatcctttccatcgcctcgacacatgtagaaaacgtaatttttttaaaattaa
aaaatatcgattgcgttttcgaaaaaattcttataagatacaagtatcaacaataaaagttaatttaagaaaggcacgaatttatatgaagagattag
atagtatagtagagttcgtgagtggttcaccgcaatttagaattatagaagtgtttgatgataaagcaccactttacacctattatggacaacaaga
tattgaagatgatttggttggtattgattctaatggtagtgacagcaaacaagtacgaaccttggataaggtcagtacgttatgtcaaggggatatt
gtttttagtttgatatcgggaaaatctactatggttggtgtgaagcatcagggatatctatacacgcaaaactacgttaaattagtaactgatgaaaa
aattgagtcaaaatatcttgtttacttacttaatgaagataaattcattaataaacagtttcaaatgggactacaggggtctcaagttctaaaatacac
actaaaacaagtgaaagaacttgagctaccagatctgcctataatagaaaaacagcgcataatcggtgaactctatttcaaccaattgcgacta
gaagcattgaaaattagagcggcaaatttggaaactatcataggggccagtttgttgaagattagatgctataattgttattaaaaggattgaagg
atgcttaggaagacgagttattaatagctgaataagaacggtgctctccaaatattcttatttagaaaagcaaatctaaaattatctgaaaaggga
atgagaatagtgaatggaccaataataatgactagagaagaaagaatgaagattgttcatgaaattaaggaacgaatattggataaatatgggg
atgatgttaaggctattggtgtttatggctctcttggtcgtcagactgatgggccctattcggatattgagatgatgtgtgtcatgtcaacagagga
agcagagttcagccatgaatggacaaccggtgagtggaaggtggaagtgaattttgatagcgaagagattctactagattatgcatctcaggtg
gaatcagattggccgcttacacatggtcaatttttctctattttgccgatttatgattcaggtggatacttagagaaagtgtatcaaactgctaaatcg
gtagaagcccaaacgttccacgatgcgatttgtgcccttatcgtagaagagctgtttgaatatgcaggcaaatggcgtaatattcgtgtgcaagg
accgacaacatttctaccatccttgactgtacaggtagcaatggcaggtgccatgttgattggtctgcatcatcgcatctgttatacgacgagcgc
ttcggtcttaactgaagcagttaagcaatcagatcttccttcaggttatgaccatctgtgccagttcgtaatgtctggtcaactttccgactctgaga
aacttctggaatcgctagagaatttctggaatgggattcaggagtggacagaacgacacggatatatagtggatgtgtcaaaacgcataccatt
ttgaacgatgacctctaataattgttaatcatgttggttacgtatttattaacttctccgagtatgctcctcaattgagtgtgatatactcatttagatgtc
tatgcttaacatatactgccgtttgaactacgttgcttaaatatgttatatgtgaagaagaagcaaggacatgaacaggttagatttaccattttggg
accaaagatttgtcctggtatttatagaggagtttttgatatgacgaataatttttggcgtgatttgccacgaccttttttttatactggcaccgatggaa
gatgtgacggatgttgtgtttcgccatgtcgtaagtgaagcagccagaccggatgtgttttttacggagtttgcgaatacagagagttattgtcac
ccggaggggaacaaaagtgtacgcgggcgtttgacttttacagaggatgaacagcccatggtggcccatatctggggagataagccggaat
atttccgtgaaatgagcatcggtatggcgaaagaaggcttcaaaggcatcgatattaatatgggttgtcccgtagcgaatgtagcccagaatgg
gaagggaagcggtctgatctgccgtcccgaaatcgcagcggatatcattcaggccaccaaagccgggggactgcccgtgagtgtaaaaac
aaggctcggtttcactaccgtggacgaatggcgcgactggttgacccatattttgaaacaagacattgtgaatttgtccattcatctgcggacaa
gagaggaaatgagcaaagtagacgcccactgggaactgattccggagattaagaaacttcgtgatgaggtggcaccagatacactgctgac
cattaacggagatattccggaccgtcagaccggcttgaagctcgctgagcaatatggagtggatggtattatgattgggcgtggtattttccaga
atccatttgcgtttgagaaggagcggaaggaacatagcagtgaagaattgcttgaactgctgcggcttcatttagatctccatgatcaatattcag
cgcaggagccacgttcgttcagccctctcgcccgcttcttcaaaatatatgtccgtggattccgggggcaagtgaactcagaaacagcttaat
gaacgccaaatcaacaagtgaagtgcgtacattgcttgatgagtttggaagcaaggatcatgatgaggtagaggaacgtggaaattaaattcc
caagttg

P12

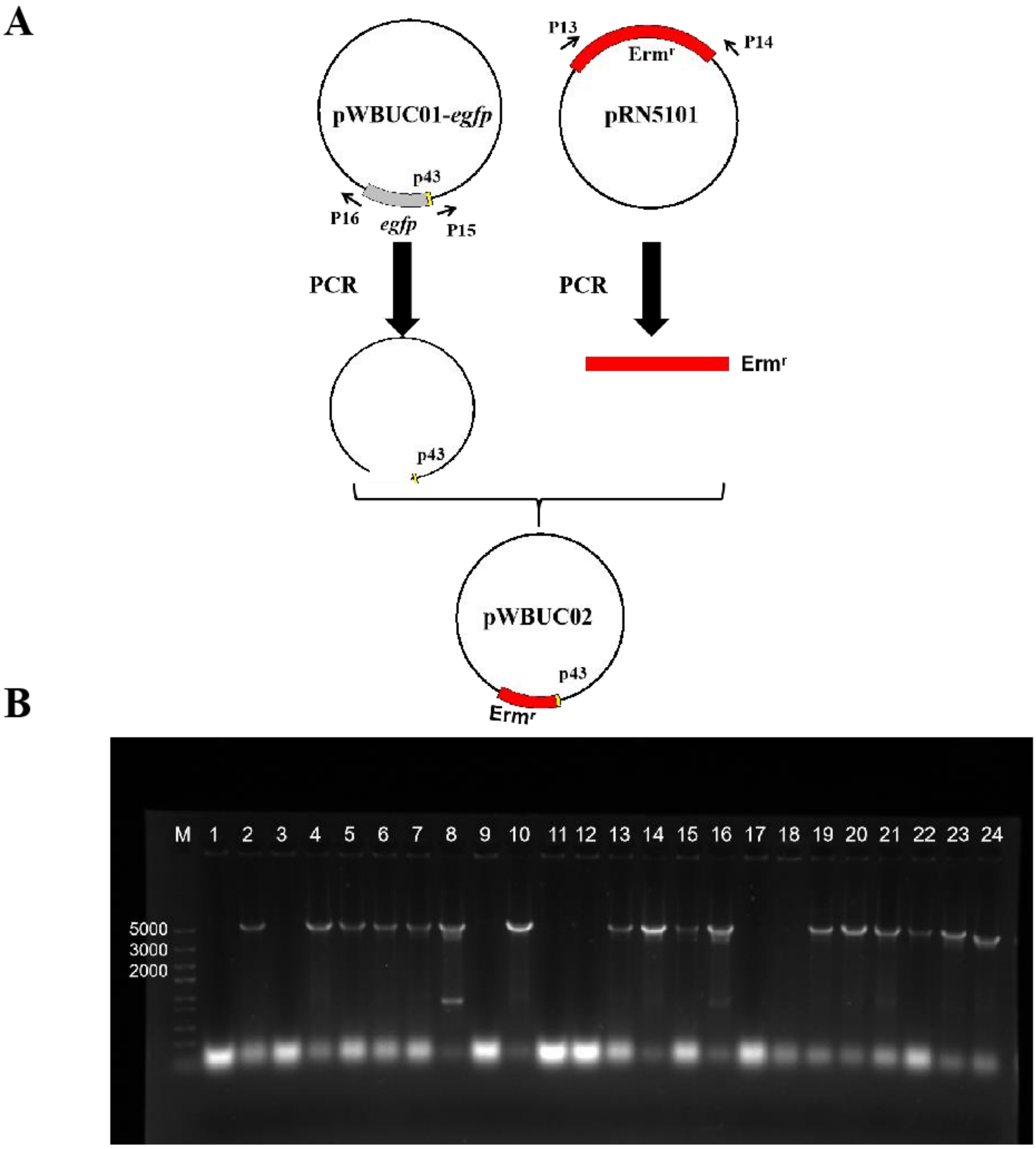

Figure S13 Construction of pWBUC02. The DNA fragment containing the erythromycin resistance gene amplified from pRN5101 was ligated with the linearized pWBUC01-*egfp* with the ORF of *egfp* deleted by using the One Step Cloning Kit (Vazyme Biotech Co., Ltd) (A). The recombinant plasmid pWBUC02 was examined with a pair of checking primers. Lane M : *Trans*5K DNA Maker; Lanes 1-23 : transformants containing pWBUC02 as templates; Lanes 24 : pWBUC01-*egfp* as the template.

SEQ. 4: Sequence of pWBUC02

tcgcgcgtttcggtgatgacggtgaaaacctctgacacatgcagctcccggagacggtcacagcttgtctgtaagcggatgccgggagcaga
caagcccgtcagggcgcgtcagcgggtgttggcgggtgtcggggctggcttaactatgcggcatcagagcagattgtactgagagtgcacc
atatgcggtgtgaaataccgcacagatgcgtaaggagaaaataccgcatcaggcgccattcgccattcaggctgcgcaactgttgggaagg
gcgatcggtgcgggcctcttcgctattacgccagctggcgaaagggggatgtgctgcaaggcgattaagttgggtaacgccagggttttccc
agtcacgacgttgtaaaacgacggccagtgaattagaactcggtacgcgcggatcttccagagattatcctctagagtcgacctgcagatcctc
tagagtcgacctgcaggcatgcaagctagcttcagcacaattccaagaaaaacacgatttagaacctaaaaagaacgaatttgaactaactcat
aaccgagaggtaaaaaaagaacgaagtcgagatcagggaatgagtttataaaataaaaaaagcacctgaaaaggtgtctttttttgatggttttg
aacttgttctttcttatcttgatacatatagaaataacgtcatttttattttagttgctgaaaggtgcgttgaagtgttggtatgtatgtgttttaaagtattg
aaaacccttaaaattggttgcacagaaaaaccccatctgttaaagttataagtgactaaacaaataactaaatagatgggggtttcttttaatattat
gtgtcctaatagtagcatttattcagatgaaaaatcaagggttttagtggacaagacaaaaagtggaaaagtgagaccatggagagaaaagaa
aatcgctaatgttgattactttgaacttctgcatattcttgaatttaaaaaggctgaaagagtaaaagattgtgctgaaatattagagtataaacaaa
atcgtgaaacaggcgaaagaaagttgtatcgagtgtggttttgtaaatccaggctttgtccaatgtgcaactggaggagagcaatgaaacatgg
cattcagtcacaaaaggttgttgctgaagttattaaacaaaagccaacagttcgttggttgtttctcacattaacagttaaaaatgtttatgatggcg
aagaattaaataagagtttgtcagatatggctcaaggatttcgccgaatgatgcaatataaaaaaattaataaaaatcttgttggttttatgcgtgca
acggaagtgacaataaataataaagataattcttataatcagcacatgcatgtattggtatgtgtggaaccaacttattttaagaatacagaaaact
acgtgaatcaaaaacaatggattcaattttggaaaaaggcaatgaaattagactatgatccaaatgtaaaagttcaaatgattcgaccgaaaaat
aaatataaatcggatatacaatcggcaattgacgaaactgcaaaatatcctgtaaaggatacggattttatgaccgatgatgaagaaaagaattt
gaaacgtttgtctgatttggaggaaggtttacaccgtaaaaggttaatctcctatggtggtttgttaaaagaaatacataaaaaattaaaccttgatg
acacagaagaaggcgatttgattcatacagatgatgacgaaaaagccgatgaagatggattttctattattgcaatgtggaattgggaacggaa
aaattattttattaaagagtagttcaacaaacgggccagtttgttgaagattagatgctataattgttattaaaaggattgaaggatgcttaggaaga
cgagttattaatagctgaataagaacggtgctctccaaatattcttatttagaaaagcaaatctaaaattatctgaaaagggaatgagaatagtga
atggaccaataataatgactagagaagaaagaatgaagattgttcatgaaattaaggaacgaatattggataaatatggggatgatgttaaggct
attggtgtttatggctctcttggtcgtcagactgatgggccctattcggatattgagatgatgtgtgtcatgtcaacagaggaagcagagttcagc
catgaatggacaaccggtgagtggaaggtggaagtgaattttgatagcgaagagattctactagattatgcatctcaggtggaatcagattggc
cgcttacacatggtcaatttttctctattttgccgatttatgattcaggtggatacttagagaaagtgtatcaaactgctaaatcggtagaagcccaa
acgttccacgatgcgatttgtgcccttatcgtagaagagctgtttgaatatgcaggcaaatggcgtaatattcgtgtgcaaggaccgacaacattt
ctaccatccttgactgtacaggtagcaatggcaggtgccatgttgattggtctgcatcatcgcatctgttatacgacgagcgcttcggtcttaact
gaagcagttaagcaatcagatcttccttcaggttatgaccatctgtgccagttcgtaatgtctggtcaactttccgactctgagaaacttctggaat
cgctagagaatttctggaatgggattcaggagtggacagaacgacacggatatatagtggatgtgtcaaaacgcataccattttgaacgatgac
ctctaataattgttaatcatgttggttacgtatttattaacttctcctagtattagtaattatcatggctgtcatggcgcattaacggaataaagggtgtg
cttaaatcgggccattttgcgtaataagaaaaaggattaattatgagcgaattgaattaataataaggtaatagatttacattagaaaatgaaaggg
gattttatgcgtgagaatgttacagtctatcccggcattgccagtcggggatattaaaaagagtataggtttttattgcgataaactaggtttcacttt
ggttcaccatgaagatggattcgcagttctaatgtgtaatgaggttcggattcatctatgggaggcaagtgatgaaggctggcgctctcgtagta
atgattcaccggtttgtacaggtgcggagtcgtttattgctggtactgctagttgccgcattgaagtagagggaattgatgaattatatcaacatatt
aagcctttgggcattttgcaccccaatacatcattaaaagatcagtggtgggatgaacgagactttgcagtaattgatcccgacaacaatttgatt
agctttttcaacaaataaaaagctaaaatctattattaatctgttcagcaatcgggcgcgattgctgaataaaagatacgagagacctctcttgtat
ctttttattttgagtggttttgtccgttacactagaaaaccgaaagacaataaaaattttattcttgctgagtctggctttcggtaagctagacaaaac
ggacaaaataaaaattggcaagggtttaaaggtggagatttttgagtgatcttctcaaaaaatactacctgtcccttgctgatttttaaacgagca
cgagagcaaaaccccccctttgctgaggtggcagagggcaggtttttttgtttctttttctcgtaaaaaaaagaaaggtcttaaaggttttatggtttt
ggtcggcactgaattcgagctcagcattattgagtggatgattatattccttttgataggtggtatgttttcgcttgaacttttaaatacagccattgaa
catacggttgatttaataactgacaaacatcaccctcttgctaaagcggccaaggacgctgccgccggggctgtttgcgtttttgccgtgatttcg **P_{43}**
tgtatcattggtttacttatttttttgccaaagctgtaatggctgaaaattcttacatttattttacatttttagaaatgggcgtgaaaaaaagcgcgcga

ttatgtaaaatataaagtgatagcggtaccaggagggctggaagaagcagaccgctaacacagtacataaaaaaggagacatgaacgcgtg
tgctctacgaccaaaactataaaacctttaagaactttcttttttacaagaaaaaagaaattagataaatctctcatatcttttattcaataatcgcatc
cgattgcagtataaatttaacgatcactcatcatgttcatatttatcagagctcgtgctataattatactaattttataaggaggaaaaaatatgggca
tttttagtatttttgtaatcagcacagttcattatcaaccaaacaaaaaataagtggttataatgaatcgttaataagcaaaattcatataaccaaatta
aagagggttataatgaacgagaaaaatataaaacacagtcaaaactttattacttcaaaacataatatagataaaataatgacaaatataagatta
aatgaacatgataatatctttgaaatcggctcaggaaaaggccattttacccttgaattagtaaagaggtgtaatttcgtaactgccattgaaatag
accataaattatgcaaaactacagaaaataaacttgttgatcacgataatttccaagttttaaacaaggatatattgcagtttaaatttcctaaaaacc
aatcctataaaatatatggtaatataccttataacataagtacggatataatacgcaaaattgtttttgatagtatagctaatgagatttatttaatcgtg **Erm^R**
gaatacgggtttgctaaaagattattaaatacaaaacgctcattggcattacttttaatggcagaagttgatatttctatattaagtatggttccaaga
gaatattttcatcctaaacctaaagtgaatagctcacttatcagattaagtagaaaaaaatcaagaatatcacacaaagataaacaaaagtataatt
atttcgttatgaaatgggttaacaaagaatacaagaaaatatttacaaaaaatcaatttaacaattccttaaaacatgcaggaattgacgatttaaac
aatattagctttgaacaattcttatctcttttcaatagctataaattatttaataagtaagttaagggatgcataaactgcatcccttaacttgtttttcgtg
tgcctatttttttgtgaatcgatgataagctgtcaaacatgagaattcttgaagacgaaagggctcgagctcggtacccgggtatcctctagagtcg
acctgcagaatcgtcgaacggcaggcgtgcaaacttggcgtaatcatggtcatagctgtttcctgtgtgaaattgttatccgctcacaattccaca
caacatacgagccggaagcataaagtgtaaagcctggggtgcctaatgagtgagctaactcacattaattgcgttgcgctcactgcccgctttc
cagtcgggaaacctgtcgtgccagctgcattaatgaatcggccaacgcgcggggagaggcggtttgcgtattgggcgctcttccgcttcctcg
ctcactgactcgctgcgctcggtcgttcggctgcggcgagcggtatcagctcactcaaaggcggtaatacggttatccacagaatcaggggat
aacgcaggaaagaacatgtgagcaaaaggccagcaaaaggccaggaaccgtaaaaaggccgcgttgctggcgtttttccataggctccgc
cccccctgacgagcatcacaaaaatcgacgctcaagtcagaggtggcgaaacccgacaggactataaagataccaggcgtttccccctggaa
gctccctcgtgcgctctcctgttccgaccctgccgcttaccggatacctgtccgcctttctcccttcgggaagcgtggcgctttctcatagctcac
gctgtaggtatctcagttcggtgtaggtcgttcgctccaagctgggctgtgtgcacgaaccccccgttcagcccgaccgctgcgccttatccgg
taactatcgtcttgagtccaacccggtaagacacgacttatcgccactggcagcagccactggtaacaggattagcagagcgaggtatgtagg **ori**
cggtgctacagagttcttgaagtggtggcctaactacggctacactagaagaacagtatttggtatctgcgctctgctgaagccagttaccttcg
gaaaaagagttggtagctcttgatccggcaaacaaaccaccgctggtagcggtggtttttttgtttgcaagcagcagattacgcgcagaaaaa
aggatctcaagaagatcctttgatcttttctacggggtctgacgctcagtggaacgaaaactcacgttaagggattttggtcatgagattatcaaa
aaggatcttcacctagatccttttaaattaaaaatgaagttttaaatcaatctaaagtatatatgagtaaacttggtctgacagttaccaatgcttaatc
agtgaggcacctatctcagcgatctgtctatttcgttcatccatagttgcctgactccccgtcgtgtagataactacgatacgggagggcttacca
tctggccccagtgctgcaatgataccgcgagacccacgctcaccggctccagatttatcagcaataaaccagccagccggaagggccgagc
gcagaagtggtcctgcaactttatccgcctccatccagtctattaattgttgccgggaagctagagtaagtagttcgccagttaatagtttgcgca
acgttgttgccattgctacaggcatcgtggtgtcacgctcgtcgtttggtatggcttcattcagctccggttcccaacgatcaaggcgagttacat
gatcccccatgttgtgcaaaaaagcggttagctccttcggtcctccgatcgttgtcagaagtaagttggccgcagtgttatcactcatggttatgg **Amp^R**
cagcactgcataattctcttactgtcatgccatccgtaagatgcttttctgtgactggtgagtactcaaccaagtcattctgagaatagtgtatgcg
gcgaccgagttgctcttgcccggcgtcaatacgggataataccgcgccacatagcagaactttaaaagtgctcatcattggaaaacgttcttcg
gggcgaaaactctcaaggatcttaccgctgttgagatccagttcgatgtaacccactcgtgcacccaactgatcttcagcatcttttactttcacca
gcgtttctgggtgagcaaaaacaggaaggcaaaatgccgcaaaaaagggaataagggcgacacggaaatgttgaatactcatactcttcctttt
ttcaatattattgaagcatttatcagggttattgtctcatgagcggatacatatttgaatgtatttagaaaaataaacaaataggggttccgcgcaca
tttccccgaaaagtgccacctgacgtctaagaaaccattattatcatgacattaacctataaaaataggcgtatcacgaggccctttcgtc

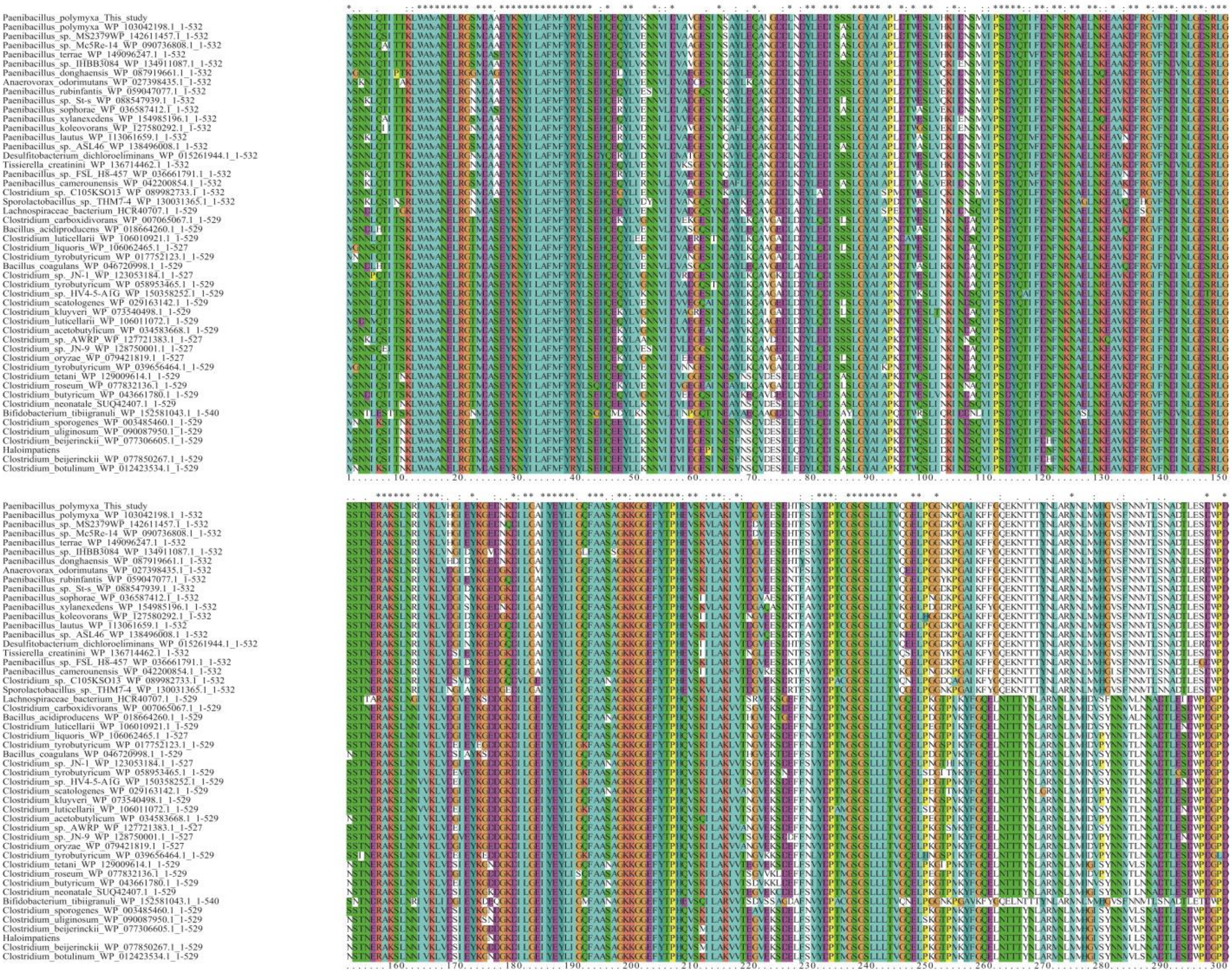

Figure S14A Alignment of M.PpoAI with ClustalX.

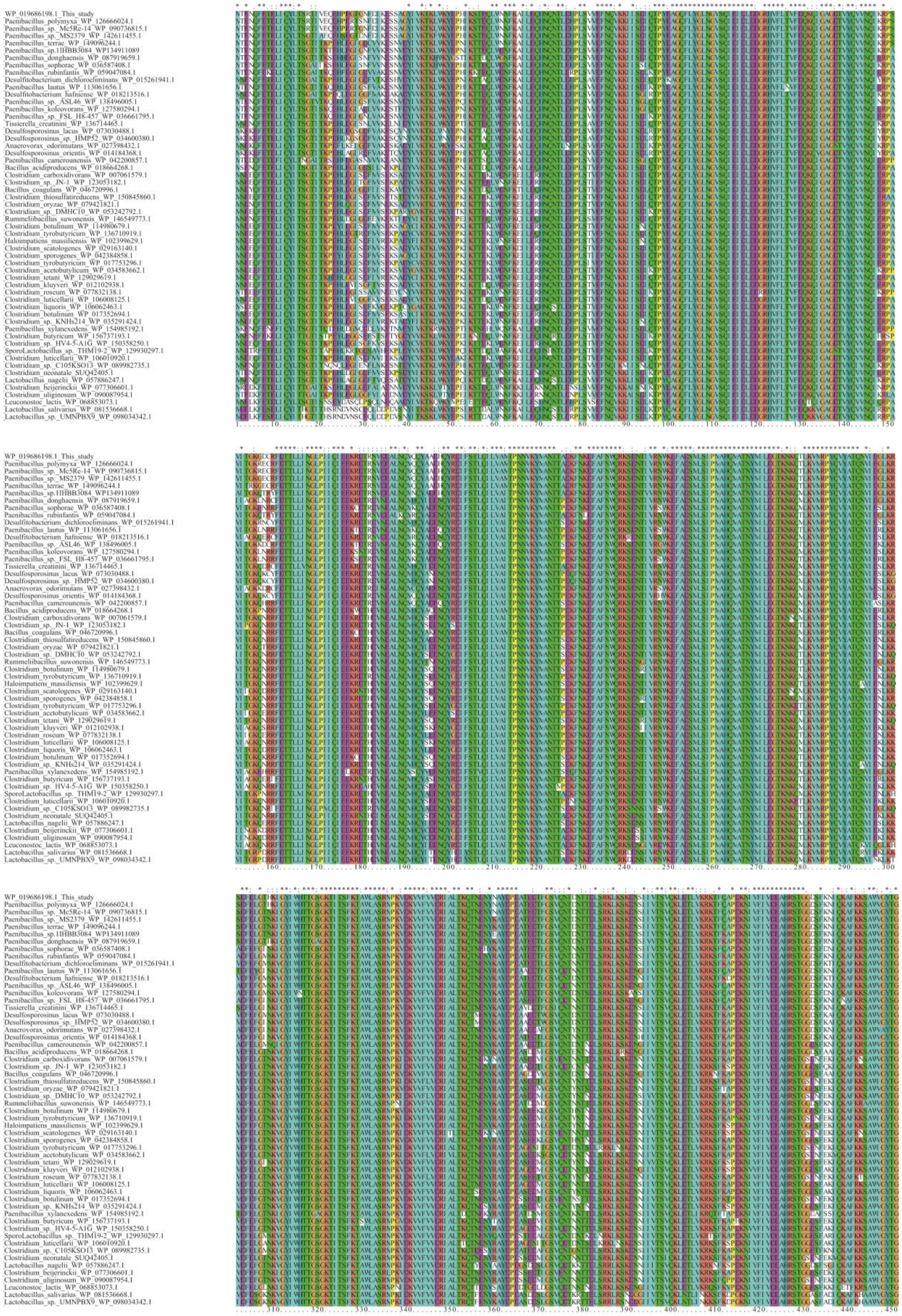

Figure S14B-1 Alignment of R.PpoAI with ClustalX.

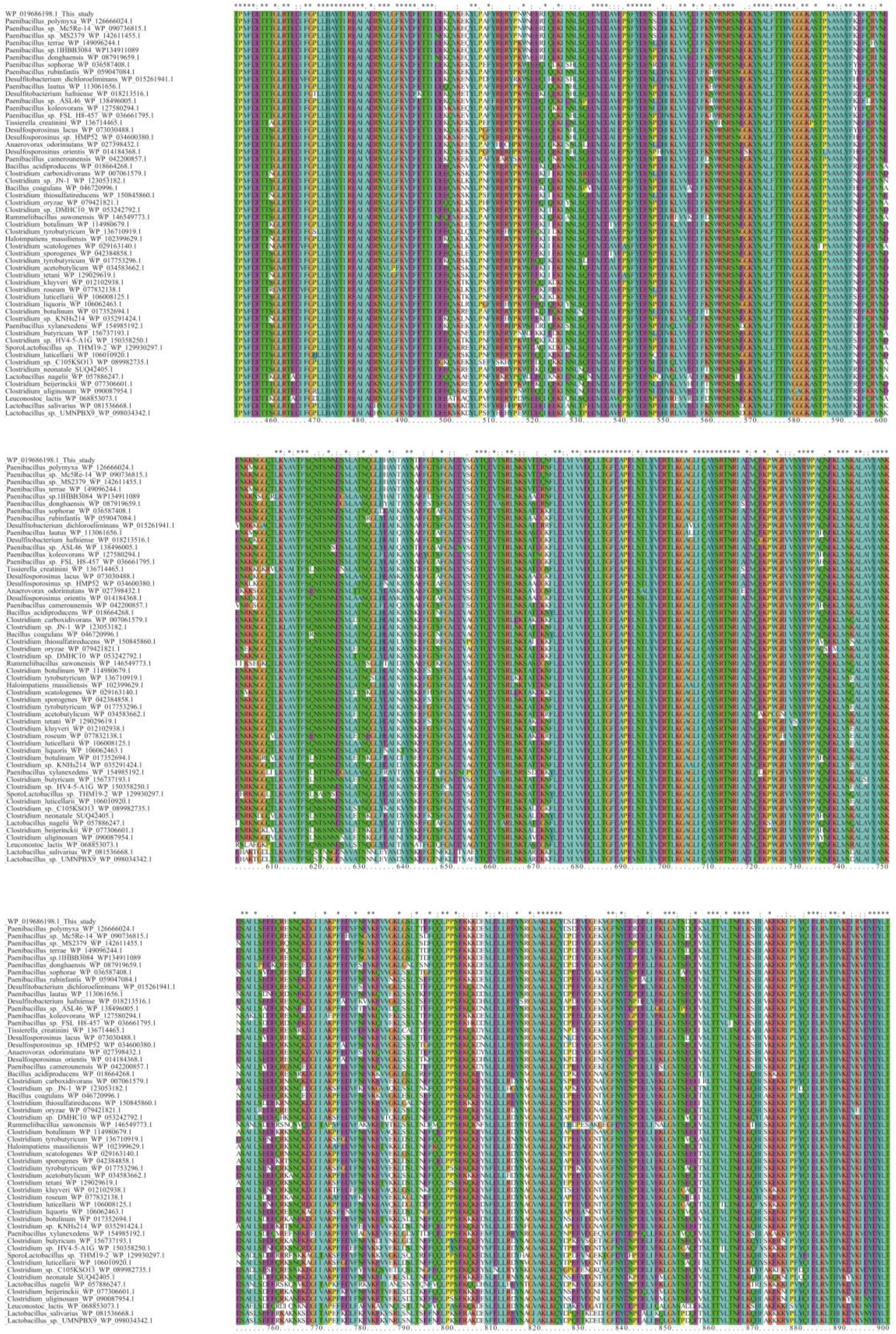

Figure S14B-2 Alignment of R.PpoAI with ClustalX

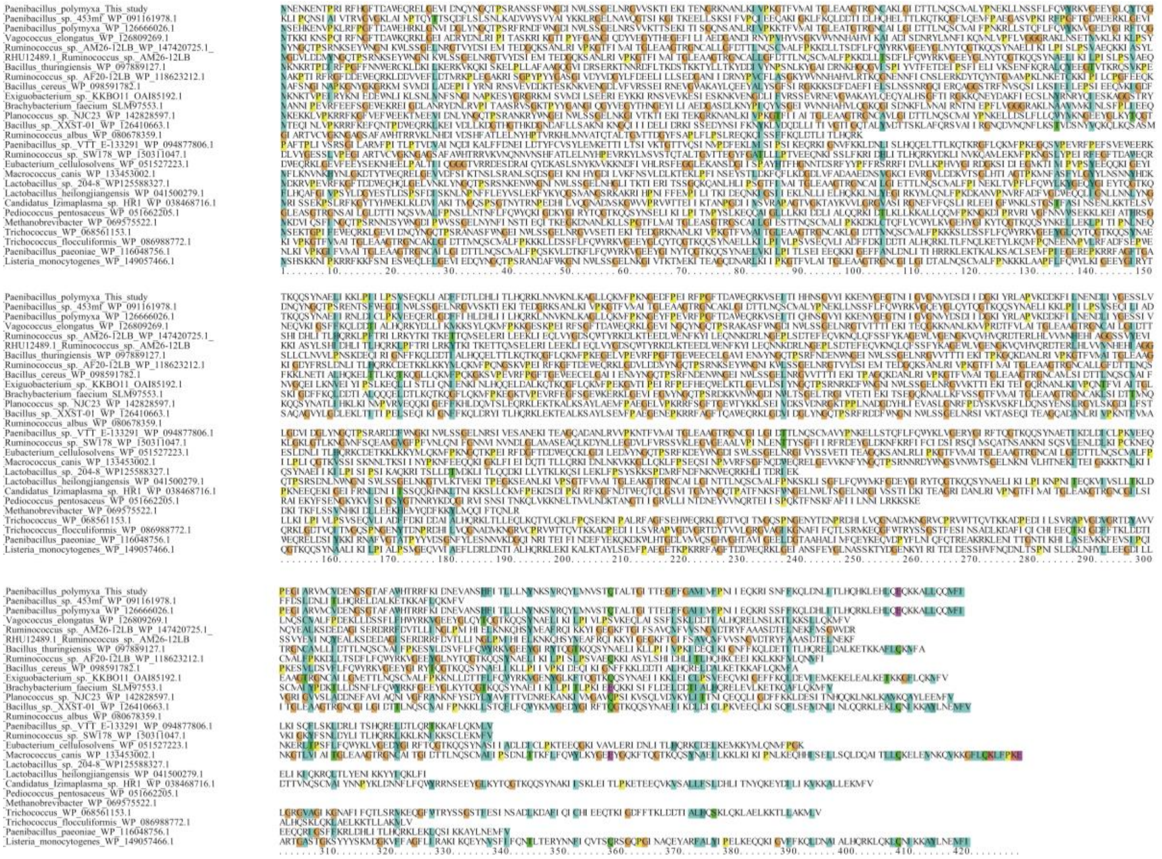

Figure S14C Alignment of S.PpoAI with ClustalX

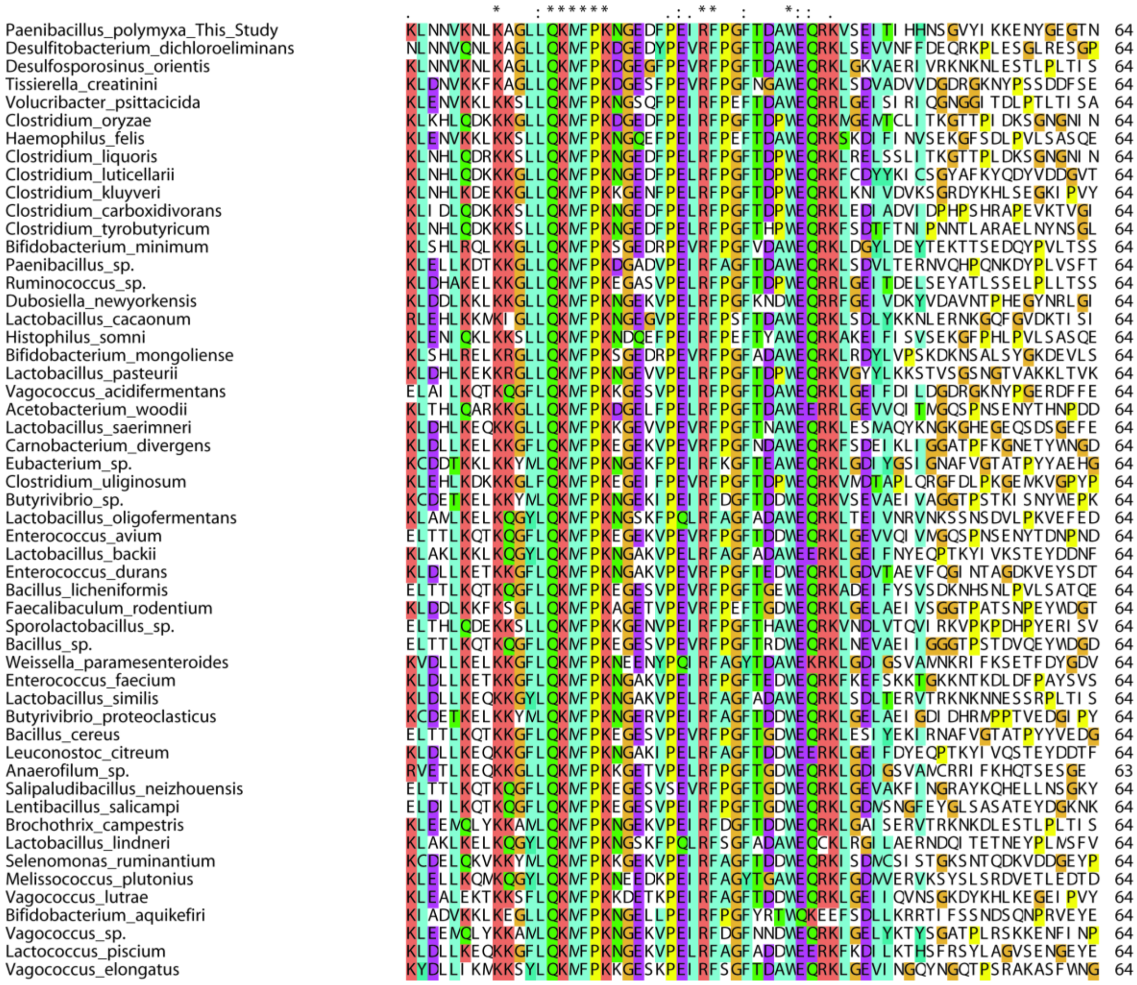

Figure S15 Alignment of CCR of S.PpoAI with ClustalX. The central conserved region (CCR) of S.PpoAI was predicted with the online software Proscan (https://www.uniprot.org/blast/). Homologous sequences were searched by BlastP of CCR of S.PpoAI against all. Sequences containing the conserved motif ‘QKMFPK’ was manually screened out. Alignment of these sequences was performed with ClustalX.

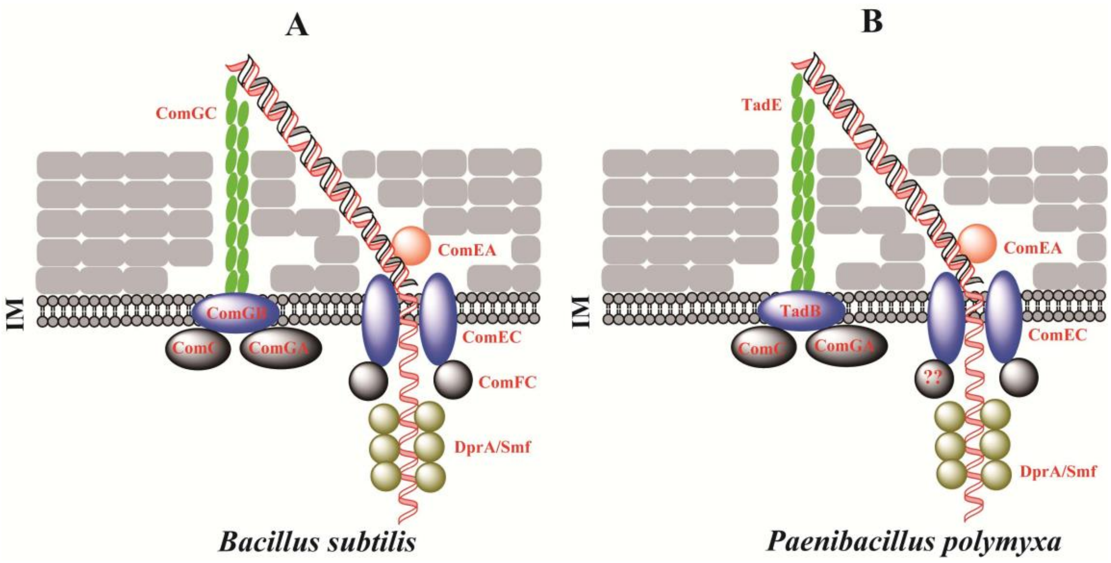

Figure S16 Diagrammatic representation of the putative DNA uptake machinery in *P. polymyxa*. A group of membrane associated proteins are involved in the uptake of DNA in *B. subtilis* (9) (A), whose orthologues in *P. polymyxa* are identified through genomic analysis (B). Although TadE and TadB are not homologous to ComGC and ComGB, components of the transformation pseudopili in *B. subtilis*, their orthologues are required for the assembly of Flp pili, which play a role similar to psudopili in natural transformation of *Micrococcus luteus* (10). Putative functions of these DNA uptake proteins were summarized in Table S8.

SUPPLEMENTARY REFERENCES

1. Sullivan MJ, Petty NK, Beatson SA. 2011. Easyfig: a genome comparison visualizer. Bioinformatics 27:1009-10.
2. Carattoli A, Zankari E, Garcia-Fernandez A, Larsen MV, Lund O, Villa L, Aarestrup FM, Hasman H. 2014. In silico detection and typing of plasmids using PlasmidFinder and Plasmid Multilocus sequence typing. Antimicrob Agents Chemoth 58:3895-3903.
3. Darling AE, Mau B, Perna NT. 2010. ProgressiveMauve: multiple genome alignment with gene gain, loss and rearrangement. PLoS One 5:e11147.
4. Shen MJ, Chen ZY, Mao XD, Wang L, Liang JY, Huo QY, Yin XY, Qiu JP, Sun DC. 2018. Two different restriction-modification systems for degrading exogenous DNA in *Paenibacillus polymyxa*. Biochem Biophys Res Commun 504:927-932.
5. Anagnostopoulos C, Spizizen J. 1961. Requirements for transformation in *Bacillus subtilis*. J Bacteriol 81:741-6.
6. Isenberg H. 2004. McFarland Standards. Clinical microbiology procedures handbook. ASM Press, Washington, DC 2:5.14.11–5.14.14.
7. Zhang W, Ding Y, Yao L, Liu K, Du B. 2013. Construction of gene knock-out system for *Paenibacillus polymyxa* SC2. Acta Microbiol Sinica 53:1258-66.
8. Wang G, Lu X, Zhu Y, Zhang W, Liu J, Wu Y, Yu L, Sun D, Cheng F. 2018. A light-controlled cell lysis system in bacteria. J Ind Microbiol Biotechnol 45:429-432.
9. Dubnau D, Blokesch M. 2019. Mechanisms of DNA uptake by naturally competent bacteria. Annu Rev Genet 53:217-237.
10. Angelov A, Bergen P, Nadler F, Hornburg P, Lichev A, Ubelacker M, Pachl F, Kuster B, Liebl W. 2015. Novel Flp pilus biogenesis-dependent natural transformation. Front Microbiol 6:84.
